# An aromatic amino acid and associated helix in the C-terminus of the potato leafroll virus minor capsid protein regulate systemic infection and symptom expression

**DOI:** 10.1371/journal.ppat.1007451

**Published:** 2018-11-15

**Authors:** Yi Xu, Washington Luis Da Silva, Yajuan Qian, Stewart M. Gray

**Affiliations:** 1 Section of Plant Pathology and Plant-Microbe Biology, School of Integrated Plant Science, Cornell University, Ithaca, NY, United States of America; 2 Institute of Biotechnology, Zhejiang University, Hangzhou, China; 3 Emerging Pest and Pathogens Research Unit, USDA, ARS, Ithaca, NY, United States of America; University of California, Davis Genome Center, UNITED STATES

## Abstract

The C-terminal region of the minor structural protein of potato leafroll virus (PLRV), known as the readthrough protein (RTP), is involved in efficient virus movement, tissue tropism and symptom development. Analysis of numerous C-terminal deletions identified a five-amino acid motif that is required for RTP function. A PLRV mutant expressing RTP with these five amino acids deleted (Δ5aa-RTP) was compromised in systemic infection and symptom expression. Although the Δ5aa-RTP mutant was able to move long distance, limited infection foci were observed in systemically infected leaves suggesting that these five amino acids regulate virus phloem loading in the inoculated leaves and/or unloading into the systemically infected tissues. The 5aa deletion did not alter the efficiency of RTP translation, nor impair RTP self-interaction or its interaction with P17, the virus movement protein. However, the deletion did alter the subcellular localization of RTP. When co-expressed with a PLRV infectious clone, a GFP tagged wild-type RTP was localized to discontinuous punctate spots along the cell periphery and was associated with plasmodesmata, although localization was dependent upon the developmental stage of the plant tissue. In contrast, the Δ5aa-RTP-GFP aggregated in the cytoplasm. Structural modeling indicated that the 5aa deletion would be expected to perturb an α-helix motif. Two of 30 plants infected with Δ5aa-RTP developed a wild-type virus infection phenotype ten weeks post-inoculation. Analysis of the virus population in these plants by deep sequencing identified a duplication of sequences adjacent to the deletion that were predicted to restore the α-helix motif. The subcellular distribution of the RTP is regulated by the 5-aa motif which is under strong selection pressure and in turn contributes to the efficient long distance movement of the virus and the induction of systemic symptoms.

## Author summary

Protein function is often dependent on its structure that is determined by the composition and chemistry of its amino acids. The C-terminal ~200 amino acids of the PLRV RTP are characteristically disordered yet this protein domain is involved in virus movement, tissue tropism and symptom development. Analysis of virus mutants, virus populations, and their cell biology allowed us to identify a short-ordered stretch of amino acids containing a conserved aromatic amino acid in the disordered RTP C-terminus. This structural feature determines efficient long-distance virus movement and symptom expression in a host-dependent manner. Virus mutants deficient in this feature generate compensatory mutations by duplication of their own sequence that restore the aromatic amino acid and the associated α-helix structural motif and in turn restore the wild-type symptom and movement phenotypes. Understanding the adaptive abilities of the virus should help to design stable virus constructs and sustainable host resistance strategies.

## Introduction

*Potato leafroll virus* (PLRV) is the type member of the *Poleroviruses*, in the family *Luteoviridae*. These viruses and the related *Luteoviruses*, collectively referred to here as luteovirids, are phloem limited viruses and transmitted by aphids in a circulative, nonpropagative manner [1, 2]. Intact virions are required to move long distance in plant hosts and aphid vectors [1, 3, 4]. In addition to the coat protein (CP) that is required for virion assembly and virus movement, there are three additional virus proteins, the minor capsid protein known as the readthrough protein (RTP), the P17 movement protein, and the P3a protein that play a role in virus movement in the plant [5–11]. P17 localizes to plasmodesmata at the companion cell-sieve element boundary and facilitates the cell-to-cell movement of assembled virus particles in a host-dependent manner [5, 8]. A small non-AUG-initiated ORF encodes the P3a protein that is required for virus long-distance movement [9]. The RTP, encoded by ORF 3 and 5, contains the 23 kDa CP and the 57 kDa read-through domain (RTD) [12]. The full-length RTP can be detected readily in infected plant tissues, but in purified virus preparations a significant portion of the C-terminal half of the RTD is proteolytically processed yielding a 51–58 kDa RTP [12, 13]. The RTD has a highly conserved N-terminal region and a variable C-terminal region. The N-terminus of the RTD is important for mediating RTP incorporation into the virion [12], and is required for aphid transmission and interaction with aphid endosymbiont proteins [14–16]. The variable C-terminal region is dispensable for aphid transmission, but it does play a role in tissue tropism, viral movement, virus accumulation, and symptom development in plant hosts [17–20]. Involvement of the full-length luteovirid RTP in virus movement was previously demonstrated by monitoring in planta progression of viral mutants either unable to synthesize the RTP or bearing small deletions in the C-terminal domain [10, 18, 19]. Rodriguez-Medina *et al* [20] reported that a C-terminal RTD deletion mutant of turnip yellows virus (TuYV-ΔRTCter) affected long-distance trafficking in a host-specific manner. In addition, while replacement of portions of the C-terminal RTD by GFP did not prevent the systemic invasion of non-inoculated leaves, virus titer was reduced and no symptoms were observed [21, 22]. This further supports the hypothesis that the C-terminal half of the RTD contains domains responsible for viral efficient infection and symptom development. The variable C-terminal region of the luteovirid RTD was determined to be highly disordered and involved in protein-protein interactions [23], but the nature of these interactions is not well studied. Several studies have reported that mutants with altered RTD function are under strong selection to acquire additional mutations that restore normal translation and function of the RTP [10, 11, 16], allowing the virus to move efficiently in phloem and accumulate to wide-type levels in plants.

Poleroviruses have monopartite, linear, single-strand RNA(+) genomes of 5.3–5.7 kb in size and they generate subgenomic RNAs (sgRNA) for expression of 3’ proximal genes [12, 24]. Three sgRNAs (sgRNA1, sgRNA2 and sgRNA3) have been described for PLRV [25]. Poleroviruses are transmitted by aphids directly into phloem tissue [1]. The virus replicates most efficiently in phloem companion cells and then moves through plasmodesmata between companion cells or into sieve elements for long distance transport [16, 26]. Plant viruses move intracellularly using the cellular cytoskeleton and/or endomembrane system to the cell periplasm and plasmodesmata [27–29]. PLRV requires assembled virions to move systemically in plants, but it is unknown if local cell-to-cell movement requires intact particles [30]. The understanding of viral movement between sieve elements and companion cells is limited, but in general virions require additional viral proteins to facilitate systemic infection of plants [31, 32] with a few notable exceptions [33–35]. Pore–plasmodesma units are specialized plasmodesmata that have branched connections with the companion cells and small pore-like openings into sieve elements [36–38]. Although the size exclusion limits of pore-plasmodesmata are greater than simple plasmodesmata connecting other cell types (e.g. mesophyll cells), they would prevent the free movement of virions and ribonucleic complexes [30, 34, 39]. *Cucumber mosaic virus* (CMV) infection can enable phloem loading and long-distance trafficking of GFP [40], and the CMV 3a movement protein fused to GFP does traffic through pore-plasmodesmata [41]. *Tobacco mosaic virus* (TMV) can also enhance its access to the phloem of mature plant tissues through the targeted disruption of auxin/indole acetic acid (Aux/IAA) transcriptional regulators that control expression of host genes involved in virus cell-to-cell movement, plasmodesmata gating [42]. These findings provide experimental evidence that virus infection can alter the size exclusion limit for plasmodesmata interconnecting the sieve element–companion cell complex. Once viruses enter the phloem, they are passively carried with the flow of photoassimilates and unload in sink tissues, apparently without the same level of control that accompanied their entry [43, 44].

Here we further investigated the functional role of the C-terminal domain of the RTP *in planta* and identified an important conserved aromatic amino residue in a conserved α-helix structural motif. These structural features, located near the C-terminus of the RTD of all PLRV isolates, are critical for RTP function. Naturally occurring pseudo-reversions of mutations that disrupt this domain restored protein structure and functions associated with phloem loading.

## Results

### A domain in the C-terminus of the PLRV RTD is involved in systemic movement and symptom development

**Fig 1:**
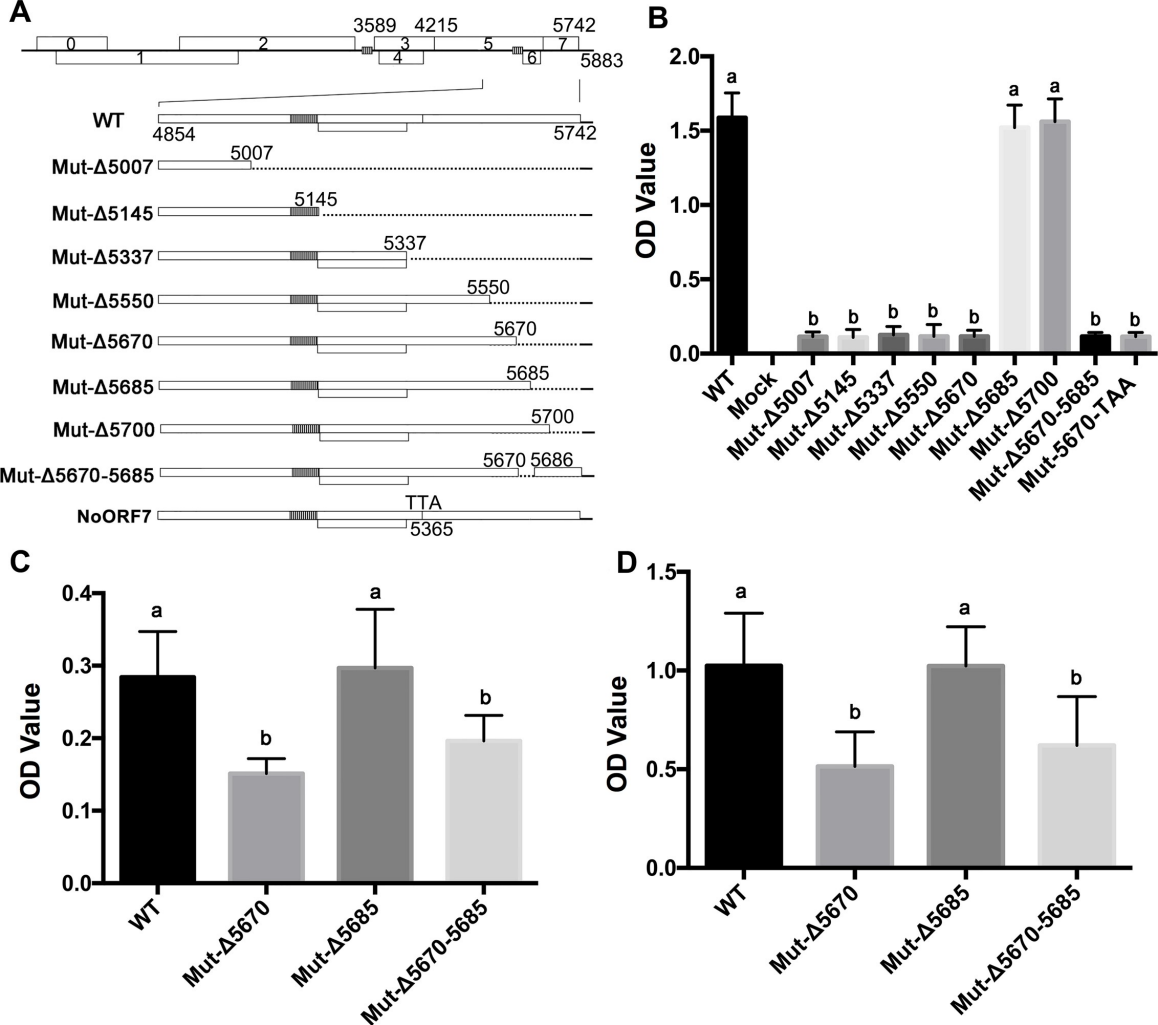
The effect of C-terminal RTP mutants on the accumulation of virus in different host plants. (A) Genome organization of PLRV and a description of the readthrough deletion mutants used in this study. (B) Virus titer measured by DAS-ELISA at 5 wpi in three immature leaves collected from each of 15 hairy nightshade plants systemically infected with wild-type (WT) PLRV and the C-terminal mutants shown in panel A and a mutant (Mut-5670-TAA that had TAA stop codon inserted at nucleotide 5670 that would prevent the translation of the five amino acid domain. (C and D) Virus titer measured by DAS-ELISA at 5 wpi in three immature leaves collected from each of 15 *N*. *benthamiana* (C) and *P*. *floridana* (D) plants systemically infected with WT-PLRV or each of the three C-terminal mutants that defined the 5-aa motif. Letters above the bars in (B), (C), (D) indicate significant differences revealed by Dunn’s multiple comparisons test p<0.05.

The C-terminal half of the PLRV RTD has been shown to be important for virus systemic movement efficiency and symptom development [10, 26], but the specific motifs directing these activities are unknown. A series of C-terminal RTD deletion mutants were constructed in an infectious PLRV clone (Fig 1A) and inoculated into the natural host *Solanum sarrachoides* (hairy nightshade, HNS). The ability of the mutants to systemically infect and induce symptoms in HNS was quantified by DAS-ELISA (Fig 1B) and monitored by observation of interveinal chlorosis symptoms five weeks post inoculation (wpi) (S1 Fig). All PLRV C-terminal mutants were infectious and were able to systemically infect plants (Fig 1B and S1A Fig). However, mutants that included deletions of nucleotides 5671–5685 (e.g. Mut-Δ5670, Mut-Δ5670–5685) accumulated to low levels in systemically infected tissues and typical interveinal chlorosis symptoms were not observed. Deletions downstream of nucleotide 5685 (Mut-Δ5685 and Mut-Δ5700) behaved like wild-type (WT) PLRV (Fig 1B and S1A and S1B Fig). Taken together, these data defined nucleotides 5671–5685 as an essential motif responsible for efficient systemic virus accumulation and symptom development in HNS. To determine whether the nucleotides or the encoded five amino acids (aa) were important for virus function, a stop codon (TAA) was inserted after nucleotide 5670 (Mut-5670-TAA). The low virus accumulation of Mut-5670-TAA and a lack of symptoms suggested that the five amino acids (leucine, phenylalanine, glutamic acid, tyrosine and glutamine, LFEYQ), not the encoding nucleotides are important for virus function (Fig 1B and S1 Fig).

### The requirement of the 5-aa motif for PLRV infection and symptom development varies among hosts

**Fig 2:**
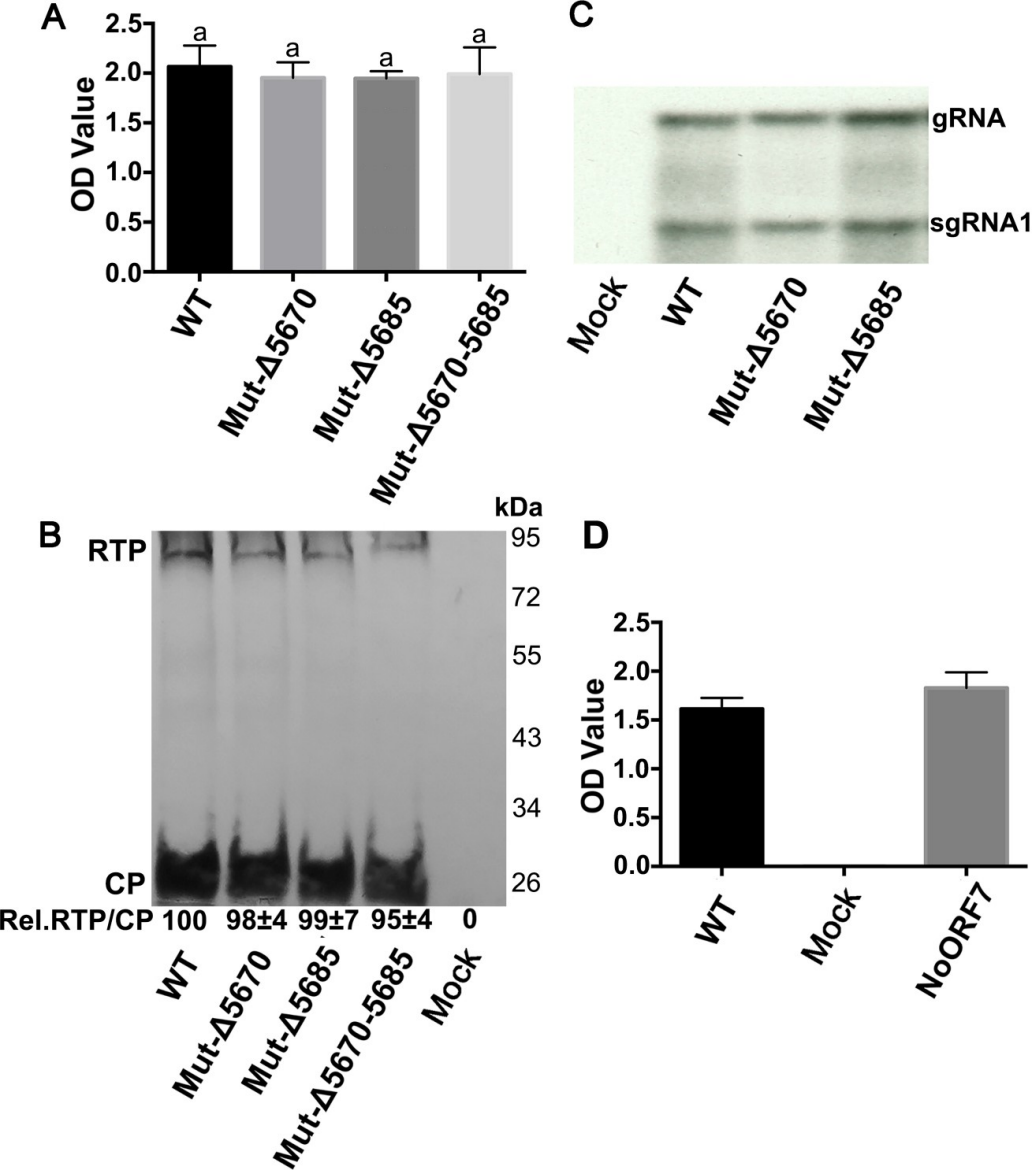
The 5-aa motif does not affect virus multiplication or readthrough protein translation. (A) Virus titer measured 3 dpi by DAS-ELISA on *N*. *benthamiana* leaves (n = 3) infiltrated with wild-type (WT) PLRV and the three C-terminal RTD mutants that defined the 5-aa domain. Letters above the bars indicate significant differences revealed by Dunn’s multiple comparisons test p<0.05. (B) Western blot analysis of total protein extracts prepared from *N*. *benthamiana* leaves (n = 3) infiltrated three days prior with wild-type (WT) PLRV, three C-terminal mutants, and agrobacterium only (mock). Signal band quantification was measured with ImageJ software (https://imagej.nih.gov/ij/). Levels of RTP (Rel. RTP/CP) detected in tissues infected with the mutants relative to WT-PLRV (set at 100) were calculated as the ratio of RTP/CP. Values represent the means (± standard error) determined from three independent experiments. (C) Northern blot analysis of total RNA extracted 3 dpi from *N*. *benthamiana* leaves (n = 3) infiltrated with WT- PLRV, two C-terminal mutants, and agrobacterium only (mock). The positions of the PLRV genomic and subgenomic RNA1 are indicated. (D) Average virus titer measured by DAS-ELISA at 5 wpi in three immature leaves collected from each of 15 hairy nightshade plants systemically infected with WT-PLRV or the NoORF7 mutant.

The role and function of some polerovirus proteins in systemic movement and virus accumulation are often host specific [8, 20, 45]. WT-PLRV, Mut-Δ5670, Mut-Δ5685, and Mut-Δ5670–5685 were individually infiltrated into the experimental host *Nicotiana benthamiana*, as well as *Physalis floridana* (groundcherry) and potato (*S*. *tuberosum* cultivar ‘Red Maria’). Virus accumulation was measured by DAS-ELISA in infiltrated leaves at 3 days post infiltration (dpi) (Fig 2) and in young systemically infected leaves at 5 wpi (Fig 1C and 1D). Virions, viral RNAs and RTP all accumulated to similar levels in the leaves infiltrated with each of the mutants (Fig 2), however, systemic infection and symptom development varied in the different hosts (Fig 1C and 1D and S1 Fig). All the mutants and WT-PLRV accumulated in systemically infected tissues of *N*. *benthamiana* and *P*. *floridana*, but similar to HNS, Mut-Δ5670 and Mut-Δ5670–5685, lacking the 5-aa motif, accumulated to significantly lower levels than WT-PLRV or Mut-Δ5685 (Fig 1C and 1D and S1A Fig). Mut-Δ5670, Mut-5670-TAA and Mut-Δ5670–5685 did not systemically infect potato (S1A Fig). The relative differences between the titers of either Mut-Δ5670 or Mut-Δ5670–5685 and WT-PLRV were less in *N*. *benthamiana*, and *P*. *floridana* than in HNS. Nevertheless, symptom expression was delayed until 6–7 wpi; two weeks after symptom expression in plants infected with WT-PLRV and the virus titer in *N*. *benthamiana*, and *P*. *floridana* plants infected with Mut-Δ5670 and Mut-Δ5670–5685 remained significantly lower than in WT-PLRV or Mut-Δ5685 infected plants (S1C and S1D Fig). The mutants all retained the expected sequence in all plants examined at 7 wpi (S1 Table). To exclude the possibility that the truncations in the C-terminus of the RTD had any effects on the production of sgRNAs 3 from which ORF 7 would be translated, real-time quantitative PCR described by Hwang *et al*., [25] was used to investigate the relative levels of PLRV sgRNAs 3 produced by WT virus and the deletion mutants. Primers for sgRNA 3 would detect genomic RNA as well as all three sgRNAs and comparable levels of RNA were detected in all samples (S1E Fig). Primers for sgRNA2 would amplify genomic RNA as well as sgRNA1 and sgRNA2, but not sgRNA3. Similar to the sgRNA3 primers, these primers detected comparable levels of RNA in all samples (S1E Fig), Taken together, these data support the conclusion that the difference in infectivity between Mut-Δ5670 and Mut-Δ5685 is not the result of altered transcription of sgRNA3 and ORF7

### The 5-aa deletion mutants move inefficiently in the phloem

**Fig 3:**
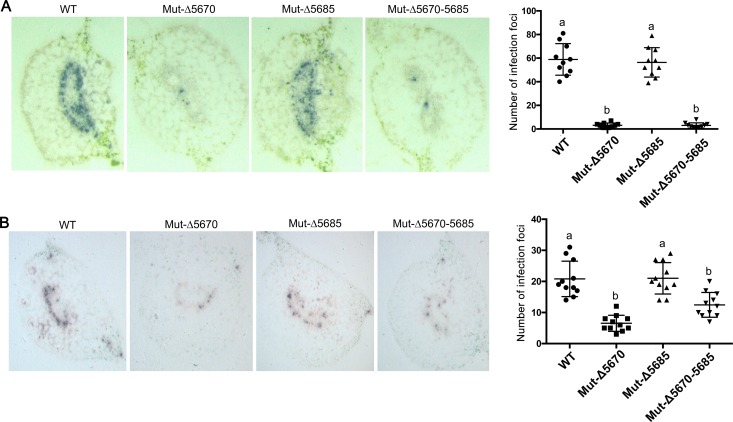
The 5-aa deletion mutants are inefficient in phloem loading. (A and B) Representative immunoprints of petiole tissue from hairy nightshade (A) and *N*. *benthamiana* (B) plants agroinoculated 5 and 7 days prior, respectively, with wild-type (WT) PLRV or readthrough protein mutants (Mut-Δ5670, Mut-Δ5685, Mut-Δ5670–5685). Tissue prints were developed with antibodies to PLRV and virus was visualized as blue-stained foci of indoxyl-precipitate. The prints were photographed under a microscope at a magnification of 50 X. The graphs to the right of the tissue prints indicate the average number and standard error of virus infection foci counted under the microscope in 10 petioles. Letters indicate significant differences as determined by Dunn’s multiple comparisons test p<0.05.

To determine if the 5-aa motif affected virus movement out of inoculated tissues, WT-PLRV, Mut-Δ5670, Mut-Δ5685, and Mut-Δ5670–5685 were individually infiltrated into HNS and *N*. *benthamiana* leaves. Tissue prints of HNS and *N*. *benthamiana* petioles from the infiltrated leaves were analyzed at 7 dpi and 5 dpi, respectively. Significantly more infection foci (>10-fold) were observed and counted in tissues of both hosts infected with WT-PLRV and Mut-Δ5685 (i.e. those containing the 5-aa motif) than in tissue infected with Mut-Δ5670 or Mut-Δ5670–5685 (Fig 3). As expected based on relative differences in virus titer between WT-PLRV and the 5-aa deletion mutants (Fig 1B and 1C), the differences in numbers of infection foci between WT-PLRV and the 5-aa deletion mutants were smaller in *N*. *benthamiana* than in HNS (Fig 3). *N*. *benthamiana* plants infiltrated with Mut-Δ5670 and Mut-Δ5670–5685 expressed mild rather than the severe interveinal chlorosis symptoms observed in plants infiltrated with WT-PLRV and Mut-Δ5685, and symptom expression was delayed 1.5–3 weeks (S1A Fig).

The nucleotides encoding the 5 aa (LFEYQ) would also be present in the ORF 7 encoded protein translated from sgRNA3. To rule out that the defect in virus accumulation and symptom development of the 5-aa deletion mutants was due to changes in the ORF 7-encoded protein rather than the RTP, we constructed a mutant (NoORF7) where the start codon of ORF 7 was eliminated by changing the “ATG” to “TTA”. The NoORF7 mutant virus accumulated to similar levels as WT-PLRV (Fig 2D) and typical interveinal chlorosis symptoms were observed by 3 wpi on HNS (S1A Fig), indicating that elimination of the ORF 7 encoded protein had no effect on virus accumulation and symptom development. The NoORF7 mutant retained the expected sequence in all plants examined at 5 wpi (S1 Table).

### Pseudo-revertants of Mut-Δ5670 are generated by a duplication of a specific genome region

**Fig 4:**
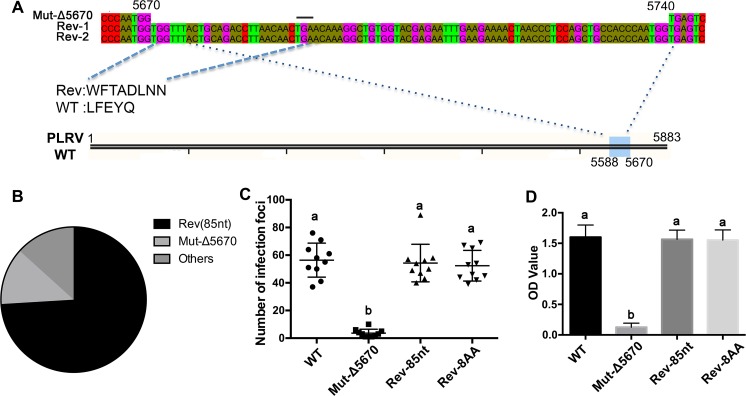
Sequence duplications within the deletion mutants can restore wild-type PLRV infection phenotypes. (A) Sequence of an 85 nucleotide insertion found between nt 5670 and 5740 in virus recovered from two of 30 plants infected with the Mut-Δ5670 that developed a WT-PLRV infection phenotype 10–12 wpi (Rev1, Rev2). The blue box on the schematic of the WT-PLRV genome indicates the origin of the 85 nucleotide duplicated sequence that was inserted after nt 5670 of the revertant viruses. The black line above the nucleotide sequence indicates a stop codon. The amino acids encoded by the insertion in the revertants (rev) and the WT virus are provided. (B) Percentage of the revertant virus population as determined by Amplicon-seq that contained either the 85 nt insertion, the original Mut-Δ5670 sequence, or other various insertions. (C) Number of virus infection foci counted 7 dpi in tissue prints from petioles of 10 leaves infected with WT-PLRV, Mut-Δ5670, a mutant with the 85 nucleotide insertion (Rev-85nt) and a mutant with a 24 nucleotide insertion (Rev-8aa) encoding the 8 amino acids defined in (A). (D) Average virus titer measured 5 wpi by DAS-ELISA in hairy nightshade leaves (n = 3) from 15 plants infiltrated with the mutants used in (C). Letters above the bars indicate significant differences as determined by Dunn’s multiple comparisons test p<0.05.

Two of 30 HNS plants infected with Mut-Δ5670, henceforth referred to as Rev1 and Rev2, developed WT-PLRV symptoms at 10–12 wpi. Virus titer in Rev 1 and Rev 2 at 12 wpi was also comparable to WT-PLRV infected plants at 12 wpi (S2A Fig). RT-PCR products spanning ORFs 5, 6, and 7, and the 3’UTR (nt 4215–5883, Fig 1A) were generated from Rev1 and Rev2, cloned into pJET1.2 vector (Fisher Scientific, USA), and sequenced. Ten clones were sequenced from each plant. All 10 clones from Rev1 and nine of 10 clones from Rev2 contained an 85-nucleotide insertion downstream of nt 5670. This insertion was a duplication of the PLRV sequence encoded by nt 5588–5670 with an additional ‘tg’ at the 5’-terminus (Fig 4A). The change in reading frame due to the addition of the “tg” resulted in the presence of a stop codon so that only 8 aa (WFTADLNN) would be added to the C-terminus of the truncated RTP (Fig 4A). To obtain high-resolution virus population information in the Rev1 and Rev2 plants, targeted amplicon sequencing technique (AmpSeq) [46] was used to investigate the sequence diversity from tissues collected at 12 wpi. We found a dominant population (75% of the sequences) that was exactly the same virus determined using Sanger sequencing (Fig 4B). The remaining population contained the original Mut-Δ5670 (12%) or insertions shorter than 85 nt, but all insertions were sequences within nt 5588–5670 (S2B Fig). In addition to the insertion, there were several single nucleotide variations (SNV) along the RTD domain that differed between the wild-type and revertant virus populations (S2C Fig). It is unknown if the SNVs in the revertant population were a result of the insertion, but those changes did not contribute to the alteration in movement or symptom expression phenotypes. In the revertant population there was a high sequence variation near nt 5670: At positions 5663 and 5671 there was a 40.0% and 83.6% C-T pyrimidine transition, respectively. At position 5672 there was a 94.31% T-G transversion.

To determine if the eight-amino acid insertion found in both Rev1 and Rev 2 could restore the WT-PLRV phenotype, the 85 nt (Rev-85nt) and the 24 nt encoding the eight-aa sequence WFTADLNN (Rev-8AA) were re-constructed in the infectious PLRV clone and infiltrated into HNS plants. The number of virus infection foci in phloem tissue, quantified in petiole tissue prints developed from tissue 7 dpi, were similar for WT-PLRV, Rev-85nt, and Rev-8AA (Fig 4C). All these viruses accumulated to similar levels, as measured by DAS-ELISA, in systemically infected HNS leaves in tissue collected at 5 wpi (Fig 4D). Furthermore, all viruses induced interveinal chlorosis in HNS at ~4 wpi and were transmitted by aphids to HNS, *P*. *floridana* and potato at similar efficiencies indicating that the revertants were biologically similar to WT-PLRV (S2 Table).

### A conserved aromatic amino acid and associated alpha helix are responsible for efficient systemic infection

**Fig 5:**
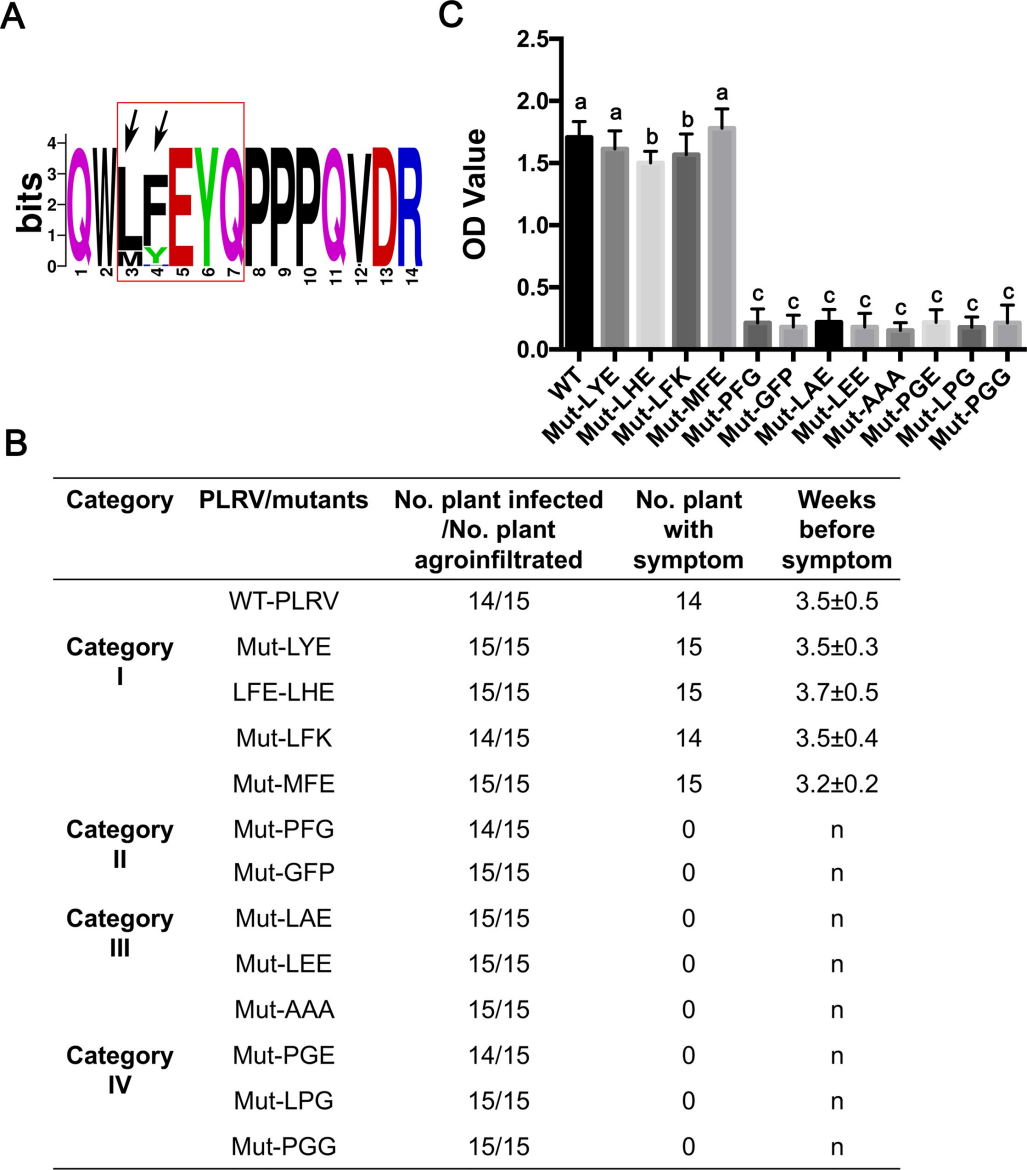
The role of aromatic residue and associated helix structure in PLRV infection of HNS. (A) Alignment of the 5-aa motifs in 32 PLRV RTD sequences available in Genbank. Each logo consists of stacks of symbols, one stack for each position in the sequence. The overall height of the stack indicates the sequence conservation at that position, while the height of symbols within the stack indicates the relative frequency of each amino at that position. The red rectangular box delineates the 5-aa motif, and the arrows indicate the position of the first two amino acids. (B) Description of helix and the aromatic residue mutants and their ability to infect and induce symptom in hairy nightshade plants (n: no interveinal chlorosis symptom). (C) Virus titer of each of the mutants was measured by DAS-ELISA at 5 wpi in immature systemically infected leaves (n = 3) from each of 15 hairy nightshade plants. Letters above the bars indicate significant differences revealed by Dunn’s multiple comparisons test p<0.05.

The C-terminus of the PLRV RTD is predicted to be highly disordered [19], however the disorder prediction algorithms IUPred and PONDR [47, 48] identified ordered domains interspersed within the disordered region of the RTD (S3 Fig). These ordered domains were predicted to be present in all the luteovirid sequences analyzed except in the enamoviruses, although the position of the ordered domains along RTD sequence varied among virus species (S3 Fig). The 5-aa LFEYQ motif in the PLRV RTD we identified as being essential for efficient systemic spread and symptom development was associated with a conserved ordered domain. Alignments of the 5-aa motifs in 32 PLRV RTD sequences available in GenBank identified conservation of the three C-terminal amino acids (EYQ), whereas there was some variation in the first two amino acids (Fig 5A). Leucine was present in the first position of 27 isolates, the remaining five had a methionine (M) in the first position. The second position was occupied by one of three aromatic amino acids; Phenylalanine (F) was found in 25 isolates whereas six isolates contained a tyrosine (Y), and one isolate contained a histidine (H) (Fig 5A). Using the protein secondary prediction algorithms: I-TASSER (https://zhanglab.ccmb.med.umich.edu/I-TASSER/) [49], RaptorX Property Prediction (http://raptorx.uchicago.edu) [50], and SPIDER2 (http://sparks-lab.org/server/SPIDER2/index.php) [51], the last short ordered domain in the RTD C-terminus that was predicted between residues 450–500 was predicted to contain three α-helices (S3 and S4 Figs). The first two residues (LF) of the 5-aa motif were located at the C-terminus of the terminal α-helix (S4 Fig). None of the variations found in the first two amino acids would be predicted to disrupt the terminal α-helix (S4 Fig). The sequence of the eight amino acid (WFTADLNN) insertion in the fully functional revertant virus was considerably different from the WT-PLRV sequence (LFEYQ), only sharing the aromatic phenylalanine (F) residue in the second position, but it was predicted to form the terminal α-helix structure similar to WT-PLRV (S4 Fig). Deletion of the LFEYQ motif (Δ5aa, S4 Fig) was predicted to completely abolish the terminal α-helix structure.

Amino acids vary in their ability to form the various secondary structure elements; regions rich in alanine (A), glutamate (E), L, and M tend to form an α-helix, while proline (P) and glycine (G) residues tend to disrupt α-helices [52–54]. To determine whether the α-helix structure or/and the aromatic amino acid is/are important for RTP function, several mutants were constructed and classified into four categories based on their amino acid composition and structure as predicted by SPIDER2 (S4 Fig). Category I mutants preserved the aromatic amino acid in position 2 and the predicted α-helix (Mut-LYE, Mut-LHE, Mut-LFK, and Mut-MFE). Category II mutants were predicted to disrupt/shorten the terminal α-helix but would retain the aromatic amino acid (Mut-PFG and Mut-GFP). Category III mutants were predicted to preserve the α-helix but would replace the aromatic amino acid (Mut-LAE, Mut-LEE, and Mut-AAA). Category IV mutants were predicted to disrupt/shorten the α-helix and would also replace the aromatic amino acid (Mut-PGE, Mut-LPG, and Mut-PGG). These 12 mutants were inoculated into HNS plants and virus systemic infection and symptom development were monitored (Fig 5B). Only mutants from category I accumulated to levels comparable to WT-PLRV at 5 wpi. Mutants in categories II, III, and IV accumulated to low virus titer levels and no symptoms were expressed even at 8 wpi (Fig 5B and 5C). All plants infected with category I mutants developed wild-type interveinal chlorosis symptoms, albeit the virus titer and the time for symptom appearance in plants varied among these mutants (Fig 5B and 5C). Viral RNA was sequenced from representative plants infected by each mutant and all original mutations were maintained in the progeny virus (S1 Table).

### The 5-aa motif affects RTP subcellular localization when co-infiltrated with PLRV

Our previous studies have shown that PLRV CP/RTP exists as multiple isoforms in plants and that RTP monomers self-interact and interact with P17 [55, 56], a host-specific PLRV movement protein [8]. To test whether the 5-aa motif was required for these interactions, bimolecular fluorescence complementation assays (BiFC) were performed in agroinfiltrated fully mature (source) and expanding (sink) *N*. *benthamiana* leaves. Both wild-type RTP and Δ5aa-RTP (expressed from Mut-Δ5670–5685) could self-interact, and the interaction signals were observed in the cytoplasm (S5A and S5B Fig). Additionally, both Δ5aa-RTP and wild-type RTP interacted with P17 in the presence or absence of PLRV clone (S5C–S5H Fig), indicating that the 5-aa motif had no observable effect on the protein-protein interactions between RTP monomers or between RTP and P17. In the absence of replicating PLRV, the fluorescent RTP or Δ5aa-RTP and P17 complexes were observed in the cytoplasm and along the periplasm (S5C–S5F Fig). In contrast, in the presence of PLRV, the fluorescent RTP-P17 complexes were observed mainly along the cell periplasm (S5G Fig) but the Δ5aa-RTP -P17 complexes were mainly in the cytoplasm (S5H Fig and S1 Movie), indicating the 5-aa may affect RTP localization. Few fluorescent inclusions were observed in sink leaves when infiltrated with Yn-RTP/Yn-Δ5aa-RTP and Yc-P17 (S5I–S5J Fig) precluding any meaningful comparisons with localization in mature leaves.

**Fig 6:**
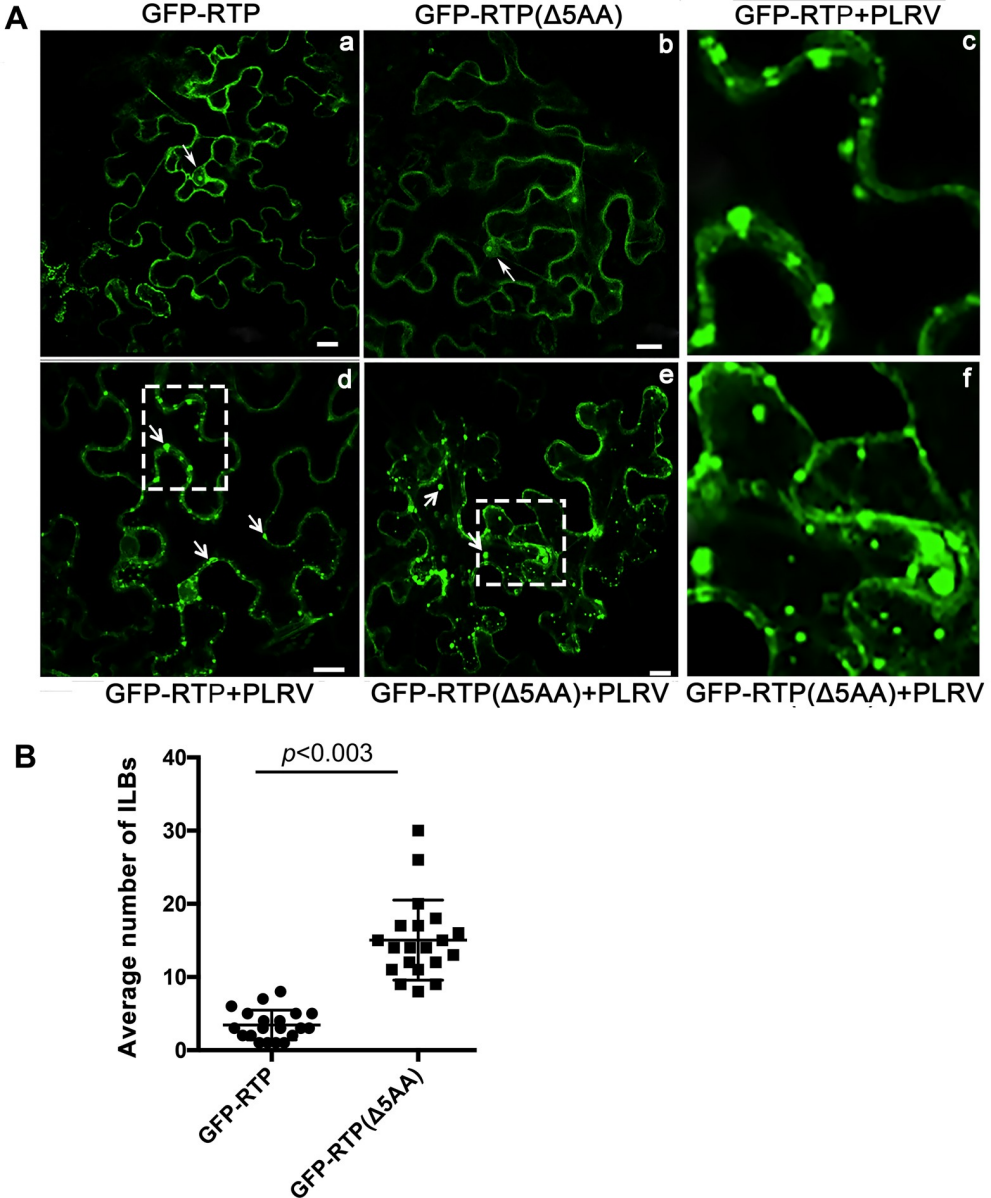
Cellular localization of GFP fluorescence infiltrated with GFP-RTP or GFP-RTP (Δ5AA) plus the infectious PLRV clone. (A) Full length WT-RTP sequence and the RTP sequence from mutant Mut-Δ5670–5685 (RTP-Δ5AA) were fused with GFP (C terminal fusion) and 35S promoter sequence and agroinfiltrated into *N*. *benthamiana* in source leaves either alone (a-b) or co-infiltrated with a full-length WT-PLRV infectious clone (d-e). GFP fluorescence was visualized by confocal microscopy 3 dpi. Arrows the nucleus (a-b) or aggregates near the cell periplasm (d-e), respectively. Bars = 10 μm. The last column shows the images of GFP-RTP+PLRV (c) and GFP-RTP(Δ5AA) +PLRV (f) magnified from white box regions in d and e, respectively. (B) The graph indicates the average number of inclusion-like bodies counted under the microscope from 30 single sections from an average of 20 cells. Membrane targeting was strictly controlled on single sections. Significance was determined by a t-test.

When expressed as a RTP-GFP fusion in *N*. *benthamiana* leaves PLRV RTP was localized in the cytoplasm and the nucleolus, but RTP lost its nucleolar localization in the presence of replicating PLRV [57]. To determine if the subcellular localization of RTP was affected by the 5aa deletion, the full-length wild-type RTP sequence and the RTP sequence from mutant Δ5670–5685 were fused with GFP (both N and C terminal fusions were constructed) and agroinfiltrated into fully mature (source) and expanding (sink) *N*. *benthamiana* leaves either alone or co-infiltrated with a full-length WT-PLRV infectious clone. These experiments were done in *N*. *benthamiana* leaves because infiltration into HNS resulted in a strong local HR reaction induced 1–2 dpi and interfered with our ability to study the effects of the PLRV mutants at the subcellular level. GFP-RTP was visualized by confocal microscopy at 3 dpi. When infiltrated independent of the infectious virus, both GFP-RTP and GFP-RTP-Δ5aa localized to the cytoplasm and nucleolus in both sink and source leaves (Fig 6A, a-b and S6B Fig, a-b). When GFP-RTP was co-inoculated into mature leaves with the full-length infectious PLRV clone it localized to the cell periplasm and formed discontinuous, variable sized inclusion-like bodies (ILBs) along the cell wall/membrane (Fig 6A, c-d, S6A Fig and S2 Movie). In contrast, when GFP-RTP-Δ5aa was co-inoculated with the infectious PLRV clone the ILBs were observed more frequently as cytoplasmic inclusions (Fig 6A, e-f, and S3 Movie). To quantify the differences, the number and location of GFP-RTP and GFP-RTP-Δ5aa ILBs were determined in at least 20 infected cells (Fig 6B). Z-Stacking of single planes through each infected cell was used to provide a composite image with a greater depth of field to better define the periplasm and a more accurate count of ILBs. Significantly more ILBs were observed in the cell cytoplasm of mature leaves co-infiltrated with GFP-RTP-Δ5aa + PLRV than those co-infiltrated with GFP-RTP + PLRV (Fig 6B). Similar to results from the BiFC assays, the low number of ILBs were observed in sink leaves co-inoculated with the full-length infectious PLRV clone and either GFP-RTP or GFP-RTP-Δ5aa (S6B Fig, c-d) precluded any meaningful analysis.

**Fig 7:**
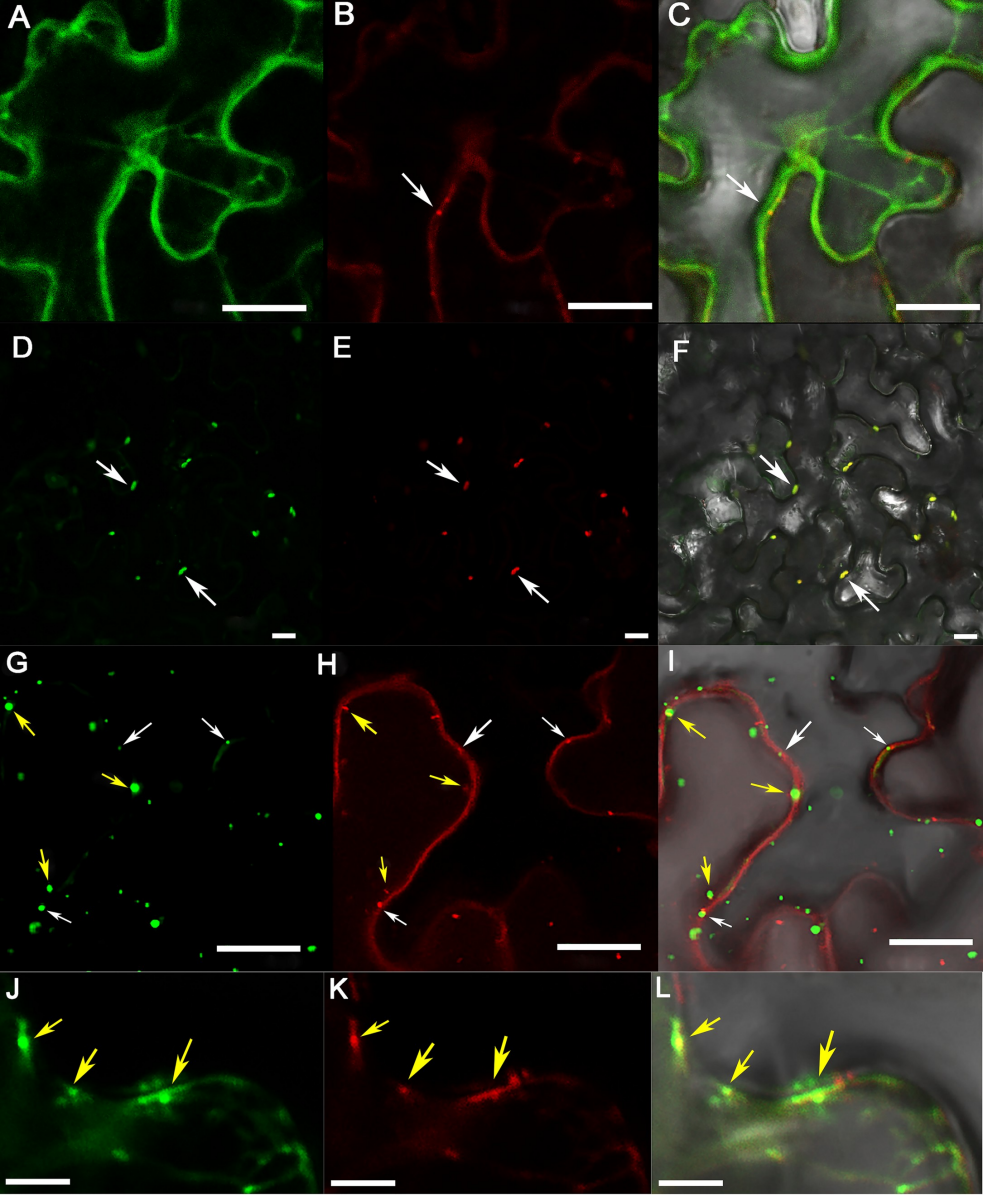
Association of RTP inclusion-like bodies (ILBs) with plasmodesmata. The first to third columns show GFP, RFP, and GFP and RFP merged image fluorescence, respectively observed in the same cells. (A-C) GFP-RTP was co-infiltrated with P17-mCherry into mature *N*. *benthamiana* leaves. A, GFP-RTP; B, P17-mCherry; C, Overlay of A and B. Arrow in B indicates P17-mCherry labeled plasmodesmata. (D-F) GFP-RTP and P17-mCherry co-infiltrated with the PLRV infectious clone into mature *N*. *benthamiana* leaves. Arrows indicate the RTP ILBs that co-localize with P17. (G-I) GFP-RTP was co-infiltrated with the PLRV infectious clone and the plasmodesmata marker mCherry-PDLP1. Yellow arrows represent the ILBs that co-localized with PDLP1-labeled plasmodesmata; white arrows represent the ILBs that are independent of PDLP1-labeled plasmodesmata. Bar = 20 μm. (J to L) Co-localization of mCherry-PDLP1 with GFP-RTP plus PLRV infectious virus in plasmolyzed cells of *N*. *benthamiana*. J, GFP-RTP; K, mCherry-PDLP1; L, Overlay of J and K. *N*. *benthamiana* cells were plasmolyzed by infiltration of 30% glycerol. Yellow arrows represent the RTP ILBs that co-localized with PDLP1-mCherry. Bar = 5 μm.

The PLRV P17 movement protein localizes to the plasmodesmata in mature tissues [58, 59]. To determine if ectopically expressed P17 could direct RTP to the cell periphery, GFP-RTP was co-infiltrated with P17-mCherry into mature leaves. P17-mCherry localized to punctate spots, presumably plasmodesmata, along the cell wall when co-infiltrated with GFP-RTP (Fig 7A–7C), but it did not redirect RTP from the cytoplasm to punctate spots along the cell wall (Fig 7A–7C). When GFP-RTP, P17-mCherry and the PLRV infectious clone were all infiltrated together, RTP and P17 did co-localized to punctate spots along the cell wall (Fig 7D–7F) suggesting that additional virus factors or changes in the cellular environment induced by the virus were needed to direct RTP or a RTP-P17 complex from the cytoplasm to the cell periphery.

### RTP inclusion-like bodies are associated with plasmodesmata

To determine if the GFP-RTP inclusions observed along the cell wall were associated with plasmodesmata, GFP-RTP and the PLRV infectious clone were co-infiltrated with the plasmodesmata marker (mCherry-PDLP1) [60]. Many of the GFP-RTP protein inclusions did co-localized with the mCherry-PDLP1 protein (Fig 7G–7I). We observed 820 GFP-RTP ILBs in 12 different fields; 230 foci (28.04%) co-localized with mCherry-PDLP1 and 422 (51.5%) were adjacent to the mCherry-PDLP1 (Fig 7G–7I). The high proportion of adjacent, but not perfectly overlapping mCherry-PDLP1 and GFP-RTP signals may be due to the dynamic nature of the GFP-RTP ILBs. Numerous inclusions were observed to traffic and accumulate together to form the ILBs (S2 Movie). We also examined the localization of mCherry-PDLP1 and GFP-RTP co-infiltrated with the PLRV infectious clone in plasmolyzed *N*. *benthamiana* cells that would distinguish between punctate spots being associated with the cell wall or cell membrane [61]. Under these conditions, most of the GFP-RTP ILBs were co-localized with mCherry-PDLP1 (Fig 7J–7L). In contrast, GFP-RTP-Δ5aa inclusions did not co-localize with the mCherry-PDLP1 protein in the presence of PLRV infectious clone (S6C Fig).

### The aromatic residue and the C-terminal helix is required for RTP localization when co-infiltrated with PLRV

**Fig 8:**
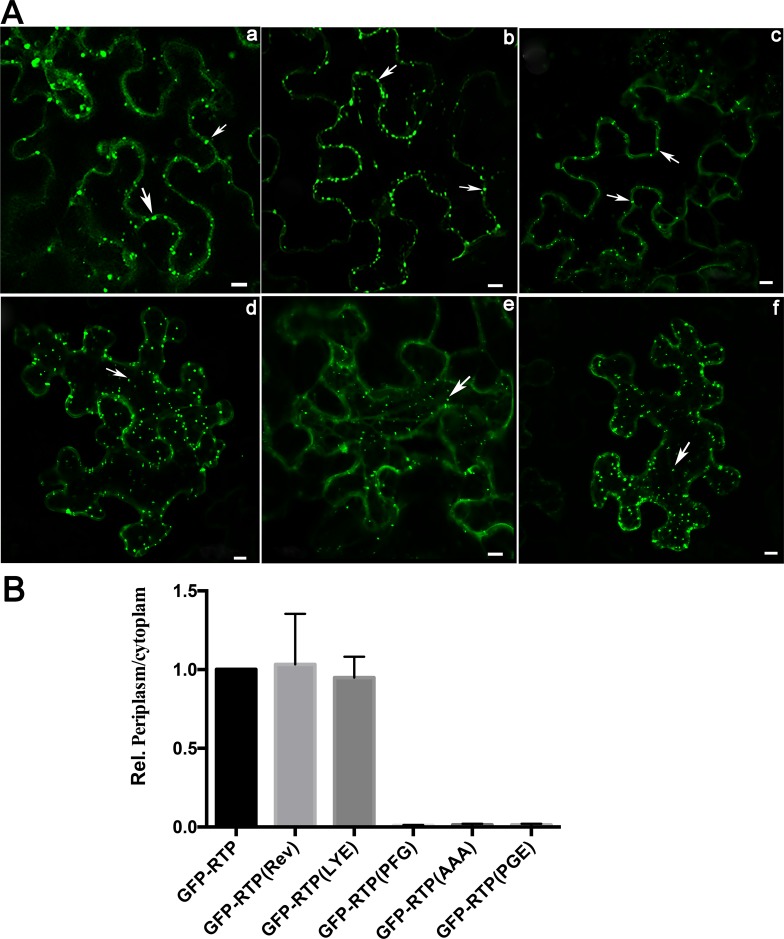
The effect of the aromatic residue and the C-terminal helix in RTP localization when agroinfiltrated with infectious PLRV. (A) Cellular localization of GFP fluorescence in mature *N*. *benthamiana* leaves agroinfiltrated with a full-length infectious PLRV clone and GFP-RTP fusions using RTP sequences from WT-PLRV (a), Rev-8AA (b), Mut-LYE (c), Mut-PFG (d), Mut-AAA (e), and Mut-PGE (f). GFP fluorescence was visualized by confocal microscopy at 3 dpi. Arrows indicate inclusion like bodies (ILBs) along the cell periplasm or in the cytoplasm. Bar = 10 μm. (B) The average number of ILBs were determined in 12 cells and counted in each of 30 single sections representing each cell. ILBs were categorized as being located along the periplasm or in the cytoplasm and the ratio of ILBs in the periplasm/cytoplasm, with that for wild-type GFP-RTP set as 1 is shown. Error bars indicate the standard deviation for 12 individual cells.

To determine whether the naturally modified RTP from the revertant virus also restored the wild-type RTP localization, the nucleotide sequence encoding WFTADLNN was cloned into the GFP expression construct in place of original amino acids (GFP-RTP-Rev) and infiltrated in *N*. *benthamiana* with infectious PLRV. GFP-RTP-Rev behaved similar to wild-type RTP-GFP with respect to subcellular localization in mature leaves (Fig 8A, a and b). To determine if the aromatic amino acid and/or helix structure were responsible for RTP localization, mutant RTPs representing each of the four categories (Mut-LYE, Mut-PFG, Mut-AAA, and Mut-PGE) were cloned into the GFP conjugated vectors and ectopically expressed with the PLRV infectious clone in mature leaves. Mutations that preserved the aromatic amino acid and the helix structure, GFP-RTP-Rev (Fig 8A and 8B) and Mut-LYE (Fig 8A, c) formed ILBs at the periplasm similar to wild-type RTP (Fig 8A, a). Mutations that replaced the aromatic amino acid and/or disrupted the helix structure (Mut-PFG, Mut-AAA, and Mut-PGE) resulted in ILBs being localized mainly in the cytoplasm (Fig 8A, d-f). These results indicate that both the aromatic residue and the C-terminal α-helix located at the C-terminus of RTD domain are required for the RTP to localize properly in the cells to facilitate systemic movement.

## Discussion

Two forms of the polerovirus RTP are found in infected plants, a full-length soluble form and a C-terminal truncated form that is incorporated into the virion [19, 26]. The RTD domain has a highly conserved N-terminal region and a variable C-terminal region. The N terminus is important for mediating RTP incorporation into the virion, aphid transmission, and interaction with aphid endosymbiont proteins [1, 2]. Whereas the variable C-terminal region is dispensable for aphid transmission, it does play a role in tissue tropism, viral movement, virus accumulation, and symptom development in plant hosts, and it can facilitate efficient aphid transmission [2, 17–20]. Furthermore, the C-terminus of RTD had no effect on RTD stability, but there are specific RNA motifs and structures located adjacent to the CP stop codon and 600 nt downstream that regulate RTP translation [62]. It was unknown whether specific RNA or protein domains or motifs can be associated with the different functions of the RTP. Here we identified that nucleotides 5671–5685 encode a protein motif near the C-terminus of the PLRV RTD that is required for efficient systemic infection and symptom development. Previous studies have reported that deletions or mutations in the RTD did not affect virus replication or accumulation in initially infected cells or at the cell level in systemically infected tissue whether the inoculum was 35S promoter-driven virus or an *in-vitro* transcript, but the number of infection foci in systemically infected tissues were reduced [11, 18, 19, 21, 62]. Our data also suggests that the 5-aa motif had no effect on virus multiplication and RTP accumulation (Fig 2). Deletion of nucleotides 5671–5685 reduced the number of infection foci in systemically infected leaves relative to WT-PLRV. This suggests that the 5-aa motif affected virus phloem loading in the inoculated leaves and/or unloading into the systemically infected tissues.

While the requirement for stable virions for systemic infection of PLRV and related luteovirids is not host specific [3, 63], movement phenotypes and the role of movement-associated virus proteins, e.g. P17 and RTP, can be host specific. A requirement for the P17 of PLRV in virus movement was different in tobacco, *P*. *floridana*, and potato [8]. Deletion of RTD C-terminus in TuYV did not affect systemic infection of *Montia perfoliata* and *A*. *thaliana*; however, the mutant virus was unable to systemically infect *N*. *benthamiana* [20]. Similarly, mutations in the RTD C-terminus of PLRV affected the ability of the altered viruses to infect four different hosts [10]. Therefore it was not surprising that we found the phloem loading phenotypes of the PLRV 5-aa mutants differed among the four hosts used in this study. The most significant effects were in the natural hosts (potato and HNS) and less so in *N*. *benthamiana* and *P*. *floridana*. The significant restriction on movement in the phloem of HNS resulted in a strong selection pressure and the generation of revertants that recovered RTP function allowing efficient movement in the phloem and systemic infection. While systemic infection was reduced in *N*. *benthamiana* and *P*. *floridana*, it was not as significant as in HNS and therefore the selection pressure to generate revertants was not likely as strong. We did not attempt to detect virus mutations in potato due to a low agroinfiltration efficiency and longer times to develop a systemic infection in the few plants that did ultimately become infected.

Naturally generated compensatory mutations have been reported from several studies when engineered deletions or mutations in the RTP coding region have negatively impacted local or systemic movement. Brault *et al*. [16], found pseudo-revertants of the *Beet western yellows virus* (BWYV) RTP restored the reading frame and the ability of the virus to move efficiently in phloem and to accumulate to wild-type levels in plants. Similarly, a mutation resulting in a frameshift and truncation of the PLRV RTD that altered the tissue tropism led to the selection of intragenic mutations or pseudorevertant mutations, either nucleotide insertions or larger deletions. All of these additional mutations resulted in an additional frameshift that restored the translation of the P5 C terminus and the WT-PLRV infection phenotype [26]. A point mutation in the BWYV RTD that impeded virus accumulation was found to either revert back to the wild-type sequence or pseudo-revert to an amino acid that was functional. Aphid transmission that was affected by other single site mutations was restored by compensatory mutations at other sites [11]. The selection of revertants or pseudo-revertants that restore virus movement and systemic infection has also been reported in several unrelated plant viruses further suggesting that movement function is under strong selection pressure. Thompson *et al*. [64], found compensatory capsid protein mutations in cucumber mosaic virus conferring systemic infectivity in *Cucurbita pepo*. Second-site reversion of a dysfunctional mutation in a conserved region of the tobacco mosaic virus movement protein was found to restore systemic infection [65]. Maize chlorotic mottle virus p31 protein, expressed as a readthrough extension of p7a, is required for efficient systemic infection. Pseudorevertants were detected to restore MCMV systemic infection when a stop codon was introduced downstream of p7a stop codon that disrupted p31 expression [66].

The variable C-termini of luteovirid RTDs are highly disordered [2, 19, 23], and therefore their structure is not well studied. We used several algorithms that independently predicted several helix structures in the C-terminus of the RTP to be conserved across all available PLRV sequences. Similar analysis of other luteovirids, except the enamoviruses which do not have the equivalent of the C-terminal RTD domain, predicted similar conserved structural motifs, albeit the positions vary among viruses (S3 and S7 Figs). The pseudo-revertants of Mut-Δ5670 that restored RTP translation and movement function were generated by duplication of sequences from a specific genome region, all of which would be predicted to recover the α-helix structure. Taken together these data support our hypothesis that the ordered α-helix structure plays an important role in RTP intrinsic functions and/or RTP-host protein interactions, and that mutations to maintain the structures will be selected. While α-helices can form helix-turn-helix motifs, leucine zipper motifs or zinc finger motifs that have particular significance in DNA binding [67–69], none of these structures were predicted in the RTD using COILS and Prabi algorithms (S8 Fig) [70]. The α-helix is also the most common structural element in transmembrane proteins [71]. The Δ5aa-RTP could not move efficiently to the cell periplasm and was sequestrated in the cytoplasm suggesting that RTP trafficking may depend on the plant endomembrane system, and the conserved C-terminal α-helix may be required for anchoring the RTP to the endomembrane. Alternatively, the α-helix may be a transmembrane domain that associates with specific lipids and this lipid-protein interaction determines the protein localization [72]. Protein structure-mediated intracellular virus trafficking is common. The NSm protein of groundnut bud necrosis virus interacts with endoplasmic reticulum membranes via a coil-coil domain at the C-terminus to remodel the endoplasmic reticulum network to form vesicles that in turn translocate NSm to plasmodesmata [73]. Deletion of 66 amino acids from the C-terminus of the tobacco mosaic virus movement protein resulted in an inability of the protein to associate with microtubules and move to plasmodesmata near the leading edge of infection [74]. Protein function is often dependent on protein structure that is determined by the composition and chemistry of its amino acids [75].

Aromatic-aromatic interactions are ubiquitous in nature and play important roles in maintaining structures of proteins [76]. For example, aromatic residues in the C-terminal helix of human apoC-I mediate phospholipid interactions and particle morphology [77]. The conserved aromatic residue in the C-terminal α-helix appears to be a particular structural feature that enables RTP to function in virus movement. The predicted structural analysis coupled with changes in biological functions we observed support a conclusion that the C-terminal domain of luteovirid RTDs would be better described as having important ordered structures at both ends with an intrinsically disordered central region. While the disordered regions will preclude structural analyses of the RTD using available technologies, we have been able to show that conserved structures in this multifunctional protein are important and this lays the groundwork for future studies. To the best of our knowledge, this is the first report that defines specific structural motifs in the C-terminus of the RTD and shows the importance of the aromatic residue in the C-terminal α-helix for PLRV phloem loading.

Poleroviruses traffic systemically as virions and the P17 movement protein does associate with the PLRV virion [3, 55]. Whether there is an association between P17 and RTP was unknown. Here we found RTP formed numerous ILBs along the cell periphery in mature source leaves, but not in developing sink leaves when co-inoculated with the PLRV infectious clone (S6A and S6B Fig). Moreover, while the RTP also interacted with P17 in the developing sink leaves in our BiFC test, the number of ILBs was much lower and the signal was less consistent than that in source leaves (S5I–S5L Fig). This is likely explained by previous research showing that P17 localizes to branched plasmodesmata in mature source leaves and roots, but not to the simple plasmodesmata found in developing sink leaves where P17 is degraded by the 26S proteasome [58, 59, 78]. Collectively, it appears that while RTP localization in mature *N*. *benthamiana* leaves requires an interaction with P17, other virus encoded factors are contributing to the subcellular localization of the RTP since ectopically expressing P17 alone was not enough to target RTP to plasmodesmata. The recently discovered polerovirus P3a protein is required for efficient virus infection [9], but its role at the cellular and subcellular level with virus infection is unknown. Alternatively, host components present in virus infected cells may play a role in facilitating P17-RTP interactions and localization. The plasmodesmal permeability is considerably down-regulated during the sink-source transition in leaves, which is accompanied by a progressive conversion from simple to branched forms of plasmodesmata. The lack of RTP localization at plasmodesmata in sink leaves suggests a differential role of the RT protein in source and sink leaves.

The manner in which poleroviruses induce interveinal chlorosis symptoms in their hosts is not well understood. In general, this type of symptom has been attributed to increased photoassimilate retention and starch accumulation in the source leaves which blocks starch transport to phloem [79]. The accumulation of virions or overexpression of virus-encoded proteins can cause a reduction in plasmodesmata permeability to photoassimilates by the excessive accumulation of callose at the plasmodesmata [18, 80, 81]. Rinne *et al*. [81], showed that constitutive expression of the tospovirus movement protein NSm in tobacco induced virus infection symptoms. This was correlated with the obstruction of NSm-targeted mesophyll plasmodesmata in source tissues by depositions of 1,3-beta-D-glucan or callose. Bruyere *et al*. [18], observed an absence of symptoms following agroinfiltration of *N*. *clevelandii* plants with BWYV containing a deletion of the C-terminal RTD region. They also reported two deletions in the N- terminus of BWYV RTD abolished symptoms. These two domains appear to correspond to the two domains we recently found to regulate RTP translation [62]. The loss of symptom expression associated with the deletions in the N-terminus of the BYWV RTD was likely an indirect effect due to a loss of RTP translation not a direct effect on symptom development. Furthermore, large deletions in the N-terminus of the PLRV RTD that do not interfere with RTP translation do not affect symptom expression (S3 Table). Our observation that PLRV RTP formed ILBs located at the cell periphery and associated with plasmodesmata in infected mature source leaves suggests that the RTP, perhaps in concert with other virus proteins, when bound at plasmodesmata can reduce the size of pore plasmodesmata units and reduce permeability to photoassimilate. ILBs comprised of RTP and other virus proteins localized at the cell periplasm and plasmodesmata of mature leaves may interact with host factors through the C-terminal domain and regulate the plasmodesmata permeability and perturbation of the symplastic connectivity for photoassimilate by inducing callose accumulation. This hypothesis is supported by a recent finding that a protein kinase binding to the C-terminal domain of the RTP of turnip yellows virus regulates virus accumulation. This protein kinase was found to localize to the plasmodesmata [20]. The composition of the RTP-associated punctate bodies associated with plasmodesmata and their role in regulating the size of pore-plasmodesmata needs to be investigated in more detail.

Both structural and non-structural forms of the RTP of *Cucurbit aphid-borne yellows virus* are essential for efficient systemic infection [19]. Here we show that in the absence of the full-length RTP, PLRV is deficient in phloem loading and systemic infection. The requirement for the C-terminal domain of the PLRV RTP for efficient long distance movement is supported further by studies on the related member of the *Luteoviridae*, *Pea enation mosaic virus* (PEMV) that lacks the homologous C-terminal part of the RTP and it is unable to move systemically in the absence of the helper umbravirus PEMV-2 [82]. Our data strongly support the hypothesis that efficient long-distance movement of poleroviruses requires the C-terminus to help with efficient phloem loading of virions. Indeed, disrupting virus phloem loading and systemic infection are common resistant strategies that plants adopt to counter virus infection [30]. Several naturally occurring host resistant genes, which restrict/promote virus phloem loading and long-distance movement have been identified [83–86]. Identifying the virus and host components involved in the interactions with the C-terminus of RTP and its role in cellular and systemic movement, along with an understanding of how the virus can compensate for perturbations of the RTP sequence should assist in developing sustainable host resistance to systemic infection.

## Materials and methods

### Plasmids generation and inoculation

Recombinant cDNA of PLRV mutants used in this study were generated using specifically designed primers according to the previously described yeast recombination method [87]. All the primers are available upon request. Briefly, by using a set of restriction enzymes, *BsrG*1 and *Xho*1 or *BsrG*1 and *Bsa*1, the plasmid pBPY, a yeast-bacteria shuttle vector containing a full-length cDNA of PLRV, was linearized and dephosphorylated with alkaline phosphatase (New England Biolabs, Ipswich, USA) to inhibit recircularization. The short insertion fragments containing the mutated region were PCR-amplified from pBPY with primers homologous to their junctions. Yeast transformation was carried out based on the LiAc/SS carrier DNA/PEG method [88]. Total DNA including the recombinant plasmid DNAs were rescued from pooled yeast colonies grown on the SD/-Trp media agar plate (Clontech, Mountain View, CA), and then used to transform *Escherichia coli* strain DH5α. Plasmid DNAs recovered from DH5α were sequenced to verify sequence integrity. *Agrobacterium tumefaciens* strain LBA4404 was transformed with plasmids extracted from *DH*5α and used for agroinfiltration of *N*. *benthamiana*, *S*. *sarrachoidies* (HNS), *P*. *floridana* and *S*. *tuberosum* as previously described [28]. To construct BiFC vectors, WT-RTP, Δ5aa-RTP and P17 were fused to the N-terminal or C-terminal fragment of YFP in the Yn and Yc vectors, respectively as described [89], and then were used for agroinfiltration into *N*. *benthamiana*. Primers used in this study are listed in S4 Table.

### Protein extraction and Western blot analysis of RTP translation

All *N*. *benthamiana* plants used in this study were grown in greenhouse maintained at 25 ± 2°C with a 16:8 h photoperiod, and 4–6 leaves were used for infiltration. Agroinfiltrated *N*. *benthamiana* leaf tissues harvested 3 days post infiltration (dpi) were ground to a powder in liquid nitrogen using a pestle and mortar; 100 mg was resuspended in 0.5ml of cold extraction buffer containing 50 mM Tris-HCl pH 6.8; 2.5% SDS; 10% glycerol and 100 mM DTT (added fresh) and incubated on ice for ~20 minutes with occasional vortexing. Samples were centrifuged, 16,000 g for 5 min, and 50 ul of the supernatant was diluted 1:1 in 2×Laemmli sample buffer (Bio-Rad, Hercules, CA) supplemented with BME (5% v/v). For Western blot, samples were incubated at 100°C for 8–10 min, separated on a 10% Mini-PROTEAN TGX precast gel (Bio-Rad), and transferred to nitrocellulose. SDS-PAGE, transfer and Western blotting were performed as described [56]. PLRV CP and RTP were detected using by using a monoclonal antibody, SCR3, that recognizes the N-terminus of the CP [90]. Blotted signal was visualized using chemiluminescence according to the manufacturer’s manual (GE Healthcare, USA) and signal band quantification was measured with ImageJ software (https://imagej.nih.gov/ij/). All experiments were repeated at least three times.

### Secondary structure prediction and amino acids analysis

Amino acids encoded from luteovirids RTP genes (ORF3/5) were used to predict the disorder domains by using the disorder prediction algorithms IUPred [48]and PONDR [47]. The protein secondary structure was predicted using I-TASSER (https://zhanglab.ccmb.med.umich.edu/I-TASSER/) [49], RaptorX Property Prediction (http://raptorx.uchicago.edu) [50], and SPIDER2 (http://sparks-lab.org/server/SPIDER2/index.php) [51]. Alignment of luteovirid sequences was performed using an online version of CLUSTALW 2.1 software with default “slow/accurate” parameters (http://www.genome.jp/tools/clustalw/). Graphical representation of the sequence conservation of 32 PLRV RTD sequences available online was generated by using the online software WebLogo [91].

### Analysis of virus in infected tissue

Virus accumulation in agroinfiltrated leaves and systemically infected plants (three leaves and fifteen plants) was detected by using double-antibody sandwich enzyme-linked immunosorbent assay (DAS-ELISA) as described previously [4]. Total RNA was extracted from *N*. *benthamiana* leaves 3 dpi as described previously [92]. PLRV gRNA and subgenomic RNA1 were detected by Northern blot using DIG Northern Starter kit per the manufacturer’s instructions (Sigma, USA) with minor modifications. RNA gel and blot analysis were performed using a protocol adapted from Xu *et al* [92]. Thirty-five micrograms of nucleic acid from each sample were separated on a 1% agarose–6% formaldehyde gel. A 300 bp DNA probe covering the PLRV genome from position 3820 to 4119 was obtained by PCR and used as template for probe labelling with Digoxigenin-dUTP. The complementary DNA strand of denatured DNA was synthesized by Klenow polymerase using the 3′-OH termini of the random oligonucleotides as primers. Progeny viruses in systemically infected plants were analyzed by sequencing reverse transcription-PCR (RT-PCR) products. Tissue immunoblot analysis was performed on petioles of leaves infiltrated with the various mutants at 7 dpi on HNS, and 5 dpi on *N*. *benthamiana* as described previously [4]. Briefly, the nitrocellulose membranes were treated with 0.2 M CaCl_2_ prior to blotting. Tissue prints were allowed to air dry and stored at 4°C until the end of the experiment, at which time the samples were analyzed under the same conditions. PLRV coat protein was detected in tissue blots using anti-PLRV immunoglobin and goat anti-rabbit immunoglobulin conjugated to alkaline phosphatase (1:5000) as secondary antibody (Promega, USA). The presence of alkaline phosphatase was detected using the 1-Step NBT/BCIP system (Pierce, Rockford, IL).

### Subcellular localization assays and confocal microscopy

To visualize the subcellular localization of RTP alone or in the presence of infectious PLRV, leaves of 6-week-old *N*. *bethamiana* plants were infiltrated with *A*. *tumefaciens* (strain GV3101) harboring either the full length GFP-RTP sequence or one of the GFP-RTP(Δ5aa) plasmids with or without co-infiltration of *A*. *tumefaciens* harboring a plasmid containing the full length PLRV clone as described previously [28]. Agrobacterium suspensions at 0.8 optical density (OD_600_) was used for infiltration. To visualize intracellular structures, the plasmodesmata marker plasmid, mCherry-PDLP1 (Sergey Ivanov et al., 2014), was transformed into *A*. *tumefaciens* GV3101 and co-infiltrated with two additional *A*. *tumefaciens* cultures transformed with GFP-RTP and the PLRV infectious clone. Leaf tissue was harvested at 48h post agroinfiltration and examined for GFP fluorescence using a Leica TCS-SP5 confocal microscope (Leica Microsystems, http://www.leica-microsystems.com/) with a 20× objective. Agroinfiltrated *N*. *benthamiana* leaves were collected at 3 dpi for GFP observation in tissues that were co-infiltrated with the fusion proteins and PLRV infectious clone. Z-stack acquisition intervals were selected to satisfy Nyquist sampling criteria. We calculated the number of spots from 30 single sections from each cell, and the membrane targeting was strictly controlled on single sections. The total number of spots was the sum of the distinct spots from each of these 30 single sections that make up the stacks. Conditions set to excite GFP and monitor the emission were as described by Ivanov *et al* [60]. Confocal images were processed using Leica LAS-AF software (Leica Microsystems). The GFP fluorophore was excited with a 488 nm laser and emission was detected at 505–545 nm. The RFP fluorophore was excited with a 532 nm laser and emission was detected at 588 nm.

### AmpSeq and data mining

The segment of the PLRV genome spanning the entire RTD domain (nt 4179–5883) was cloned by RT-PCR from WT-PLRV infected HNS and the revertant plants at 12 wpi, respectively. Library construction and next-generation sequencing were performed at the Cornell University Biotechnology Resource Center as described by Yang *et al* [46]. The raw data were aligned by Burrows-Wheeler Aligner (BWA) software [93] after quality check and PCR duplication filter. The reads were visualized by Tablet [94]. Single nucleotide variations (SNV) along the RTD domain were generated by Lofreq (version 2) using default settings [95]. Virus-derived reads from WT-PLRV and the revertant virus library were mapped to the reference genome (wild-type RTD sequence after removing PCR duplicates) to mitigate the effects of PCR amplification bias introduced during library construction.

### Real-time quantitative PCR

Real-time quantitative PCR was performed based on Hwang *et al*., [25] with modifications. Briefly, total RNA was extracted individually from *N*. *benthamiana* leaves infiltrated with WT-PLRV, Mut-Δ5670 or Mut-Δ5685 at 3 dpi by using TRIzol reagent (Invitrogen, USA) as described above. Contaminating DNA was removed by using DNase I treatment according to the manufacturer’ instructions (Ambion, USA). cDNA was synthesized using iScript cDNA Synthesis Kit (Bio-Rad, USA) according to the manufacturer’s instruction. Primers were designed using Primer Premier 6 software (Premier Biosoft, Palo Alto, USA), and PLRV sgRNA2 primer set (P2) was F: 5’-ATGCTACAGTCGATGGTACAGA-3’ and R: 5’-CTATAATAGTAGCAGCAGCGACAT-3’; PLRV sgRNA3 primer set (P3) was F: 5’-TGGAGAAGAGAGAGGAAAATGTC-3’ and R: 5’-TCTTAAGGGTCCCTCCCTCGA-3’. RT-qPCR was performed using CFX96 Touch Real-Time PCR Detection System with the SsoAdvanced Universal Probes Supermix (Bio-Rad). The *N*. *benthamiana* actin gene was used as internal standard, and 1 ng plasmid containing the full-length of PLRV infectious clone was used as control for normalization. Each experiment was performed with three biological replicates and three technical replicates.

### Aphid transmission assays

HNS plants systemically infected with WT-PLRV or the 5-aa deletion mutants were used as virus source tissue in aphid transmission assays. The tests were performed as described previously by Peter et al. [10] with the exception that eight aphids were placed onto each recipient plant. A 24 h acquisition access period was followed by a 5-day inoculation access period. The fumigated plants were placed in a greenhouse and observed for symptom expression and assayed for systemic infection by double antibody sandwich (DAS)-ELISA at 3–5 wpi.

## Supporting information

S1 Fig Ability of WT-PLRV and mutants to systemically infect and induce symptoms in different hosts. (A) Systemic infection efficiency and symptom phenotypes of various PLRV RTP mutants in four host plants, *S*. *sarrachoides* (hairy nightshade), *S*. *tuberosum* (potato), *N*. *benthamiana* (tobacco) and *P*. *floridana* (groundcherry). “n” = no symptom, and “nd” = “not detected”. (B) Interveinal chlorosis symptoms observed at 5 wpi on leaves of hairy nightshade plants infected with WT-PLRV and the RTP mutants. Virus titer was measured by DAS-ELISA at 7 wpi in three randomly selected systemically infected leaves from each of 15 *N*. *benthamiana* (C) and *P*. *floridana* (D) plants. Letters above the bars indicate significant differences revealed by Dunn’s multiple comparisons test p<0.05. (E) Transcripts expression of gRNA, sgRNA1 and sgRNA2 were detected by sgRNA2 primer set (P2); gRNA, sgRNA1, sgRNA2 and sgRNA3 transcripts were detected by sgRNA3 primer set (P3). The value in the Y-axis represents the transcripts expression relative to the 1ng plasmid containing the full-length infectious clone which contained one copy of the area of interest for amplification of each sgRNA, and the transcripts expression of gRNA, sgRNA1, sgRNA2 and sgRNA3 in WT-PLRV normalized to *N*. *benthamiana* actin was set as standard 1; tests were repeated three independent times, and each with three technical replicates.
(TIF)

S2 Fig Detection of pseudoreversions in plants that were initially infected with the RTP deletion mutant, Mut-Δ5670. (A) Comparison of virus titer, measured by DAS-ELISA, in three randomly selected systemically infected leaves collected from each of 15 hairy nightshade plants agroinoculated with wild-type (WT) PLRV, plants agroinoculated with Mut-Δ5670 that remained symptomless, or two plants agroinoculated with Mut-Δ5670 (Rev-1 and Rev-2) that expressed wild-type symptoms 10 wpi. Leaf tissue was collected at 12 wpi. (B) The five most predominant sequences after the 85nt insertion (Fig 4A) inserted at position nt 5670 in the revertant virus population. (C) Single nucleotide variants (SNV) detected by Lofreq (version 2) [95] along the nucleotide sequence encoding the readthrough protein from the wild type (WT) virus population and the revertant population. X-axis represents the position of the SNV along the coding region, the Y-axis (left) shows the percentage of variation found in the nucleotide position and Y-axis (right) represents the sequencing depth.
(TIF)

S3 Fig Prediction of the ordered/disordered regions in the RTD of luteovirids. Sequences of ORF5 (encoding the luteovirid RTD domain) were downloaded from GenBank and analyzed using the PONDR algorithm to identify ordered and disorder regions within the RTD. Arrows indicate the ordered regions within the C-terminal domain of the wild-type (WT) PLRV and revertant RTD which contains the 5-aa motif. The genome of the three enamoviruses, *Pea enation mosaic virus* (PEMV), *Citrus vein enation virus* (CVEV) and *Grapevine enamovirus* (GEV) do not contain a C-terminal domain of the RTD. X-axis represents the position along the PLRV RTD region. Y-axis (left) indicates the raw score for each predictor for each amino acid in the RTD domain. The horizontal line demarcates ordered and disordered regions and the bold horizontal lines indicate the predicted disordered regions. PLRV, *Potato leafroll virus*; PLRV-Rev is the RTD reversion mutant that contains an eight amino acid substitution for the five amino acid motif found in wild-type PLRV; CYDV, *Cereal yellow dwarf virus*; TuYV, *Turnip yellows virus*; CABYV, *Cucurbit aphid-borne yellows virus*; BYDV, *Barley yellow dwarf virus*-PAV; SbDV, *Soybean dwarf virus*.
(TIF)

S4 Fig Prediction of helix structures that are upstream of the 5-aa motif in the C-terminal RTD of WT-PLRV and various mutants. Red boxes indicate the 5aa region that regulates efficient systemic infection and symptom expression in wild-type (WT) PLRV and other RTD mutants described in the text. The aa predicted to form α-helix structures are noted by a green H below the aa designation. SEQ: the position number in the whole RTD domain; SS: secondary structure.
(TIF)

S5 Fig Detection of RTP/ RTP (Δ5aa) self-interaction and interaction with P17. WT-RTP, Δ5aa-RTP and P17 were fused to the N-terminal or C-terminal fragment of YFP in the Yn and Yc vectors and infiltrated into *N*. *benthamiana* source (A-H) and sink (I-J) leaves in various combinations. The fluorescent signals were visualized by confocal microscopy 2 dpi using the same settings for all observations. (A) Yn-RTP and Yc-RTP; (B) Yn-RTP (Δ5aa) and Yc-RTP (Δ5aa); (C) Yn-RTP and Yc-P17; (D) Yn-P17 and Yc-RTP; (E) Yn-RTP (Δ5aa) and Yc-P17; (F) Yn-P17 and Yc-RTP (Δ5aa). (G) Yn-RTP, Yc-P17 and PLRV clone. (H) Yn-RTP (Δ5aa), Yc-P17 and PLRV clone. (I) Yn-RTP and Yc-P17; (J) Yn-RTP (Δ5aa) and Yc-P17; Bar = 20 μm. Red arrows indicate the nucleus in A and B, the white arrows indicate RTP aggregates in C-J.
(TIF)

S6 Fig Cellular localization of GFP fluorescence in source and sink *N*. *benthamiana* leaves infiltrated with GFP-RTP and the infectious PLRV clone. (A) GFP-RTP co-infiltrated with the PLRV infectious clone into source (mature) leaves. Samples were observed with confocal microscopy 3 dpi and the images processed for GFP florescence (left panel), bright-field (middle panel) or merged (right panel). Arrows indicate inclusion–like bodies of varying size. Bar = 2 μm. (B) Full length wild-type RTP and the RTP-Δ5AA fused with GFP (C terminal fusion) infiltrated alone (a-b) or co-infiltrated with the PLRV infectious clone (c-d) into sink (developing) leaves of *N*. *benthamiana*. GFP fluorescence was visualized by confocal microscopy 3 dpi. a: GFP-RTP (WT); b: GFP-RTP (Δ5AA); c: GFP-RTP (WT) + PLRV; d: GFP-RTP (Δ5AA) + PLRV. Arrows in a and b indicate the nucleus and arrows in c and d indicate the occasional punctate bodies observed. Bar = 10 μm. (C) GFP-RTP (Δ5AA) and mCherry-PDLP1 co-infiltrated with the PLRV infectious clone into mature *N*. *benthamiana* leaves. Left panel, GFP-RTP (Δ5AA) and arrows represent the inclusion bodies in the cytoplasm; Middle, mCherry-PDLP1 and arrows indicate PDLP1-labeled plasmodesmata; Right panel, overlay of left and middle panels. Bar = 10 μm.
(TIF)

S7 Fig Prediction of helix structures located in the C-terminal half of the RTD of representative luteovirids. The position and aa acids predicted to form are labeled with an H in green type below the aa designation. SEQ: the position number in the whole RTD domain; SS: secondary structure.
(TIF)

S8 Fig Prediction of helix-turn-helix motifs localized in the PLRV RTD. (A) Prediction by COILS algorithm (https://embnet.vital-it.ch/software/COILS_form.html). X-axis represents the position of the amino acid along the RTP. Y-axis: high value represents helix-turn-helix motifs. (B) Prediction by Prabi algorithm (https://npsa-prabi.ibcp.fr/cgi-bin/npsa_automat.pl?page=/NPSA/npsa_hth.html). The red rectangular box showed there is no significant helix-turn-helix motif in PLRV RTD.
(TIF)

S1 Table Sequence information of WT-PLRV and mutants from constructed plasmids and cloned from infected plants. (DOCX)

S2 Table Pathogenicity and aphid transmissibility of PLRV revertants infecting hairy nightshade plants. (DOCX)

S3 Table Pathogenicity of PLRV RTD N-terminal deletion mutants infecting hairy nightshade plants. (DOCX)

S4 Table Primers used in this study. (XLSX)

S1 Movie The trafficking of inclusion-like bodies (ILBs) in the BiFC assay. 2Yn-RTP(Δ5aa), 2Yc-P17 and infectious PLRV clone were co-infiltrated into mature *N*. *benthamiana* source leaves, and the yellow fluorescence was visualized by confocal microscopy 3 dpi. Red: auto-fluorescence of chloroplasts.
(MP4)

S2 Movie The trafficking of ILBs to the cell periplasm. GFP-RTP and infectious PLRV clone were co-infiltrated into mature *N*. *benthamiana* leaves. The GFP fluorescence was visualized by confocal microscopy 3 dpi. Arrows represent the ILBs trafficking.
(MP4)

S3 Movie The trafficking of ILBs in the cytoplasm when GFP-RTP (Δ5aa) and infectious PLRV clone are co-infiltrated. GFP-RTP (Δ5aa) and infectious PLRV clone were co-infiltrated into mature *N*. *benthamiana* leaves, and the GFP fluorescence was visualized by confocal microscopy 3 dpi. Arrows represent the ILBs trafficking.
(MP4)
