# Supplementary figures and images for: An aromatic amino acid and associated helix in the C-terminus of the potato leafroll virus minor capsid protein regulate systemic infection and symptom expression
Source: PLoS Pathog. 2018 Nov 15;14(11):e1007451. doi: 10.1371/journal.ppat.1007451 (PMC6264904; doi:10.1371/journal.ppat.1007451)

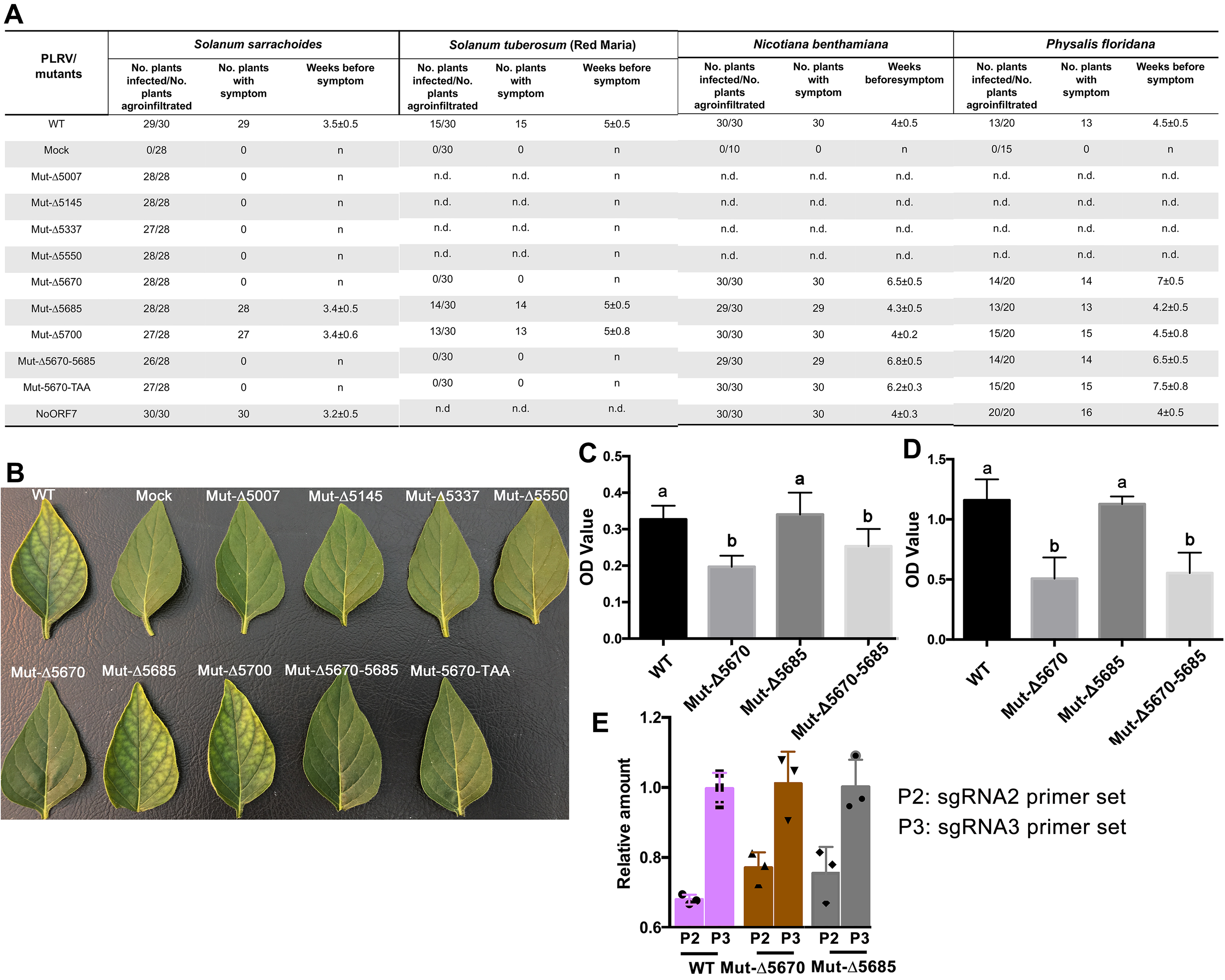

Supplement: S1 Fig — (A) Systemic infection efficiency and symptom phenotypes of various PLRV RTP mutants in four host plants, S. sarrachoides (hairy nightshade), S. tuberosum (potato), N. benthamiana (tobacco) and P. floridana (groundcherry). “n” = no symptom, and “nd” = “not detected”. (B) Interveinal chlorosis symptoms observed at 5 wpi on leaves of hairy nightshade plants infected with WT-PLRV and the RTP mutants. Virus titer was measured by DAS-ELISA at 7 wpi in three randomly selected systemically infected leaves from each of 15 N. benthamiana (C) and P. floridana (D) plants. Letters above the bars indicate significant differences revealed by Dunn’s multiple comparisons test p<0.05. (E) Transcripts expression of gRNA, sgRNA1 and sgRNA2 were detected by sgRNA2 primer set (P2); gRNA, sgRNA1, sgRNA2 and sgRNA3 transcripts were detected by sgRNA3 primer set (P3). The value in the Y-axis represents the transcripts expression relative to the 1ng plasmid containing the full-length infectious clone which contained one copy of the area of interest for amplification of each sgRNA, and the transcripts expression of gRNA, sgRNA1, sgRNA2 and sgRNA3 in WT-PLRV normalized to N. benthamiana actin was set as standard 1; tests were repeated three independent times, and each with three technical replicates. (TIF) [file ppat.1007451.s001.tif]

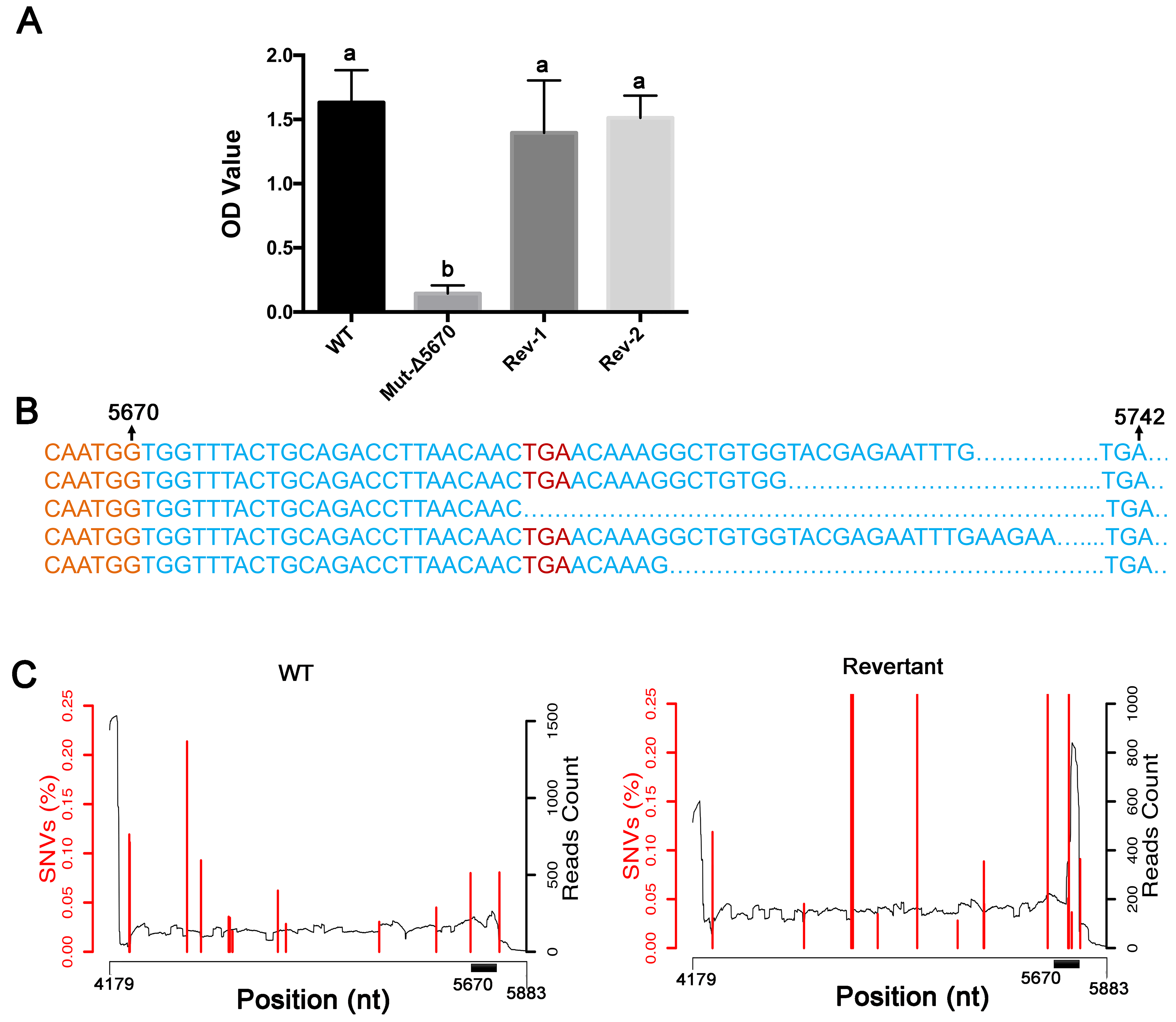

Supplement: S2 Fig — (A) Comparison of virus titer, measured by DAS-ELISA, in three randomly selected systemically infected leaves collected from each of 15 hairy nightshade plants agroinoculated with wild-type (WT) PLRV, plants agroinoculated with Mut-Δ5670 that remained symptomless, or two plants agroinoculated with Mut-Δ5670 (Rev-1 and Rev-2) that expressed wild-type symptoms 10 wpi. Leaf tissue was collected at 12 wpi. (B) The five most predominant sequences after the 85nt insertion (Fig 4A) inserted at position nt 5670 in the revertant virus population. (C) Single nucleotide variants (SNV) detected by Lofreq (version 2) [95] along the nucleotide sequence encoding the readthrough protein from the wild type (WT) virus population and the revertant population. X-axis represents the position of the SNV along the coding region, the Y-axis (left) shows the percentage of variation found in the nucleotide position and Y-axis (right) represents the sequencing depth. (TIF) [file ppat.1007451.s002.tif]

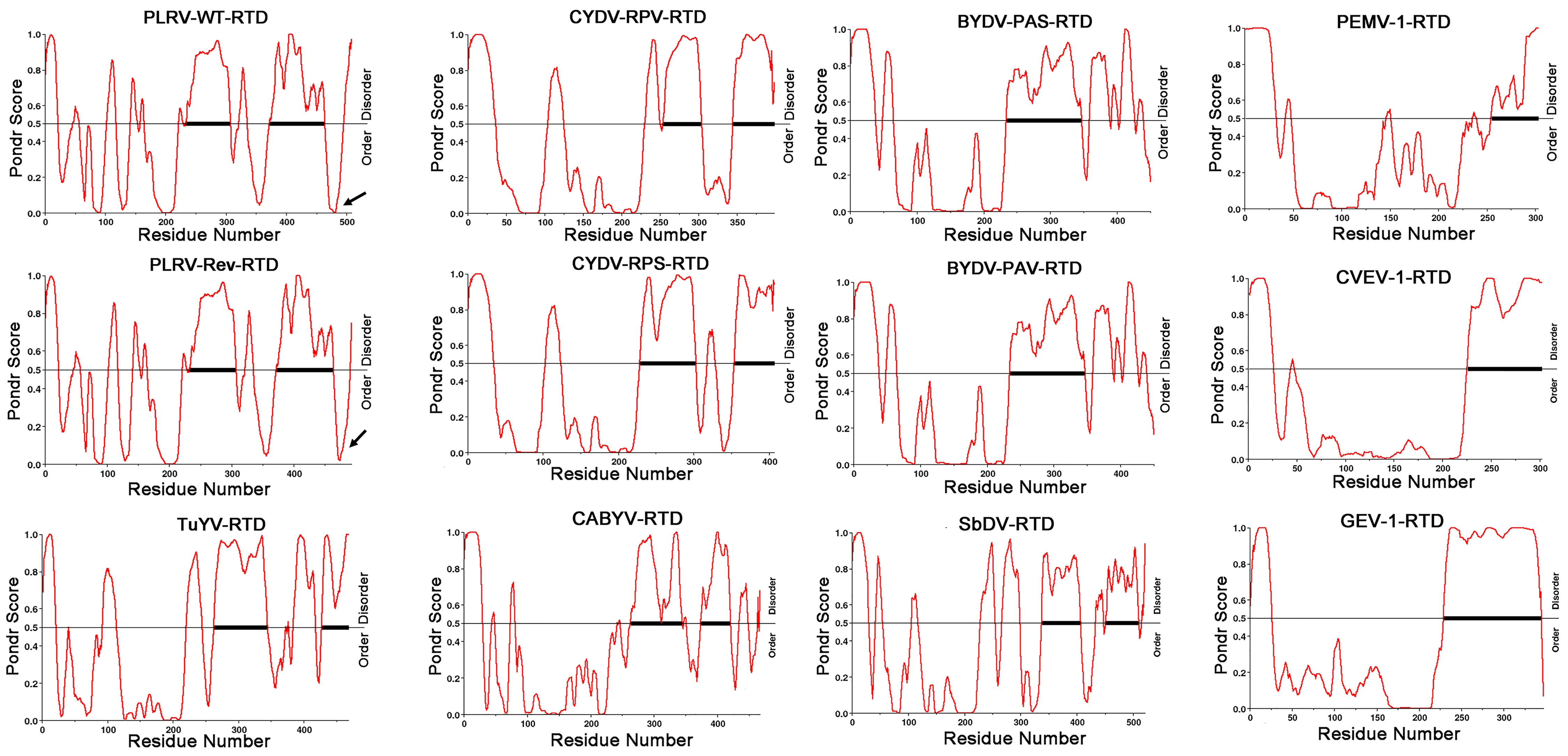

Supplement: S3 Fig — Sequences of ORF5 (encoding the luteovirid RTD domain) were downloaded from GenBank and analyzed using the PONDR algorithm to identify ordered and disorder regions within the RTD. Arrows indicate the ordered regions within the C-terminal domain of the wild-type (WT) PLRV and revertant RTD which contains the 5-aa motif. The genome of the three enamoviruses, Pea enation mosaic virus (PEMV), Citrus vein enation virus (CVEV) and Grapevine enamovirus (GEV) do not contain a C-terminal domain of the RTD. X-axis represents the position along the PLRV RTD region. Y-axis (left) indicates the raw score for each predictor for each amino acid in the RTD domain. The horizontal line demarcates ordered and disordered regions and the bold horizontal lines indicate the predicted disordered regions. PLRV, Potato leafroll virus; PLRV-Rev is the RTD reversion mutant that contains an eight amino acid substitution for the five amino acid motif found in wild-type PLRV; CYDV, Cereal yellow dwarf virus; TuYV, Turnip yellows virus; CABYV, Cucurbit aphid-borne yellows virus; BYDV, Barley yellow dwarf virus-PAV; SbDV, Soybean dwarf virus. (TIF) [file ppat.1007451.s003.tif]

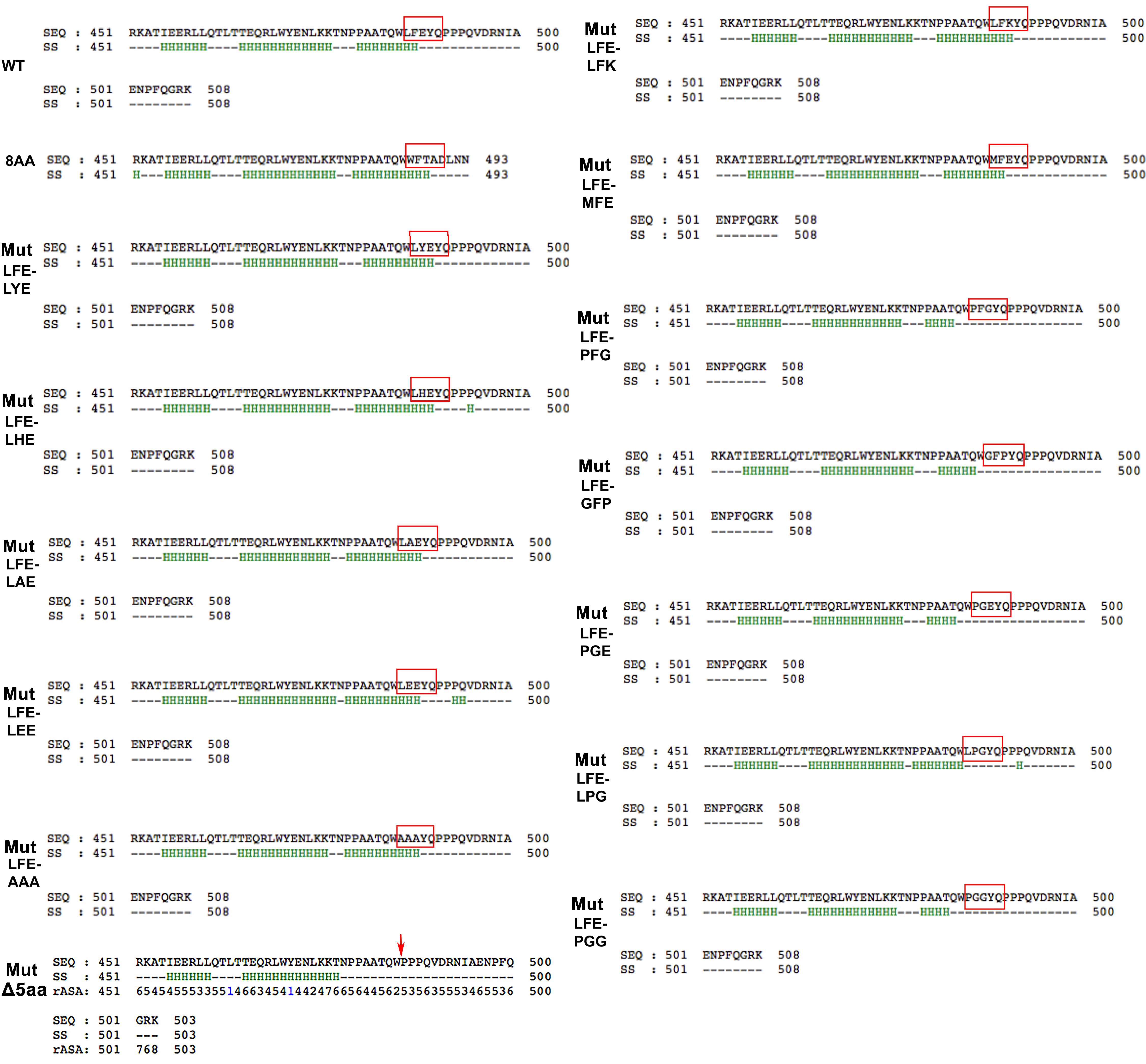

Supplement: S4 Fig — Red boxes indicate the 5aa region that regulates efficient systemic infection and symptom expression in wild-type (WT) PLRV and other RTD mutants described in the text. The aa predicted to form α-helix structures are noted by a green H below the aa designation. SEQ: the position number in the whole RTD domain; SS: secondary structure. (TIF) [file ppat.1007451.s004.tif]

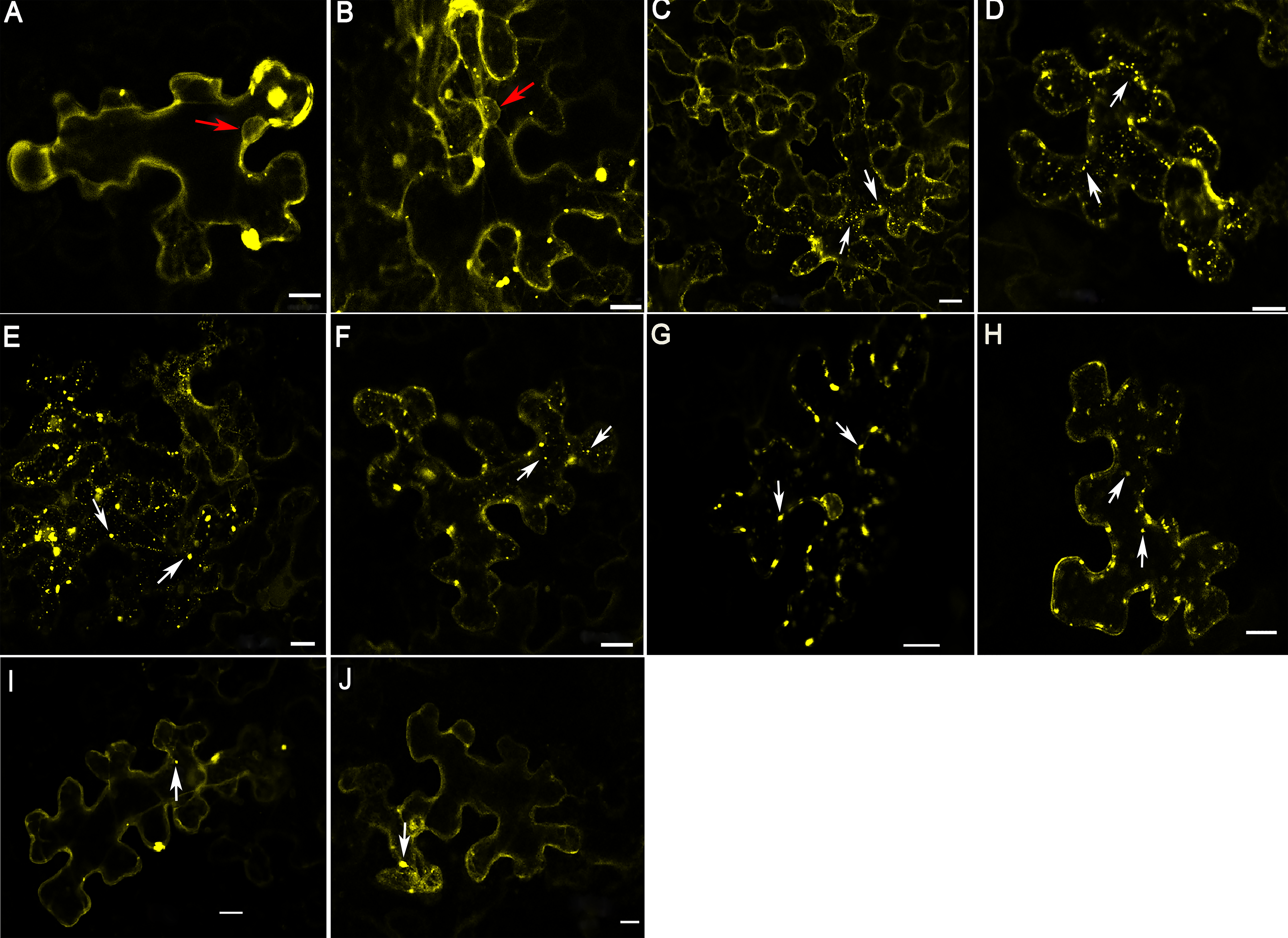

Supplement: S5 Fig — WT-RTP, Δ5aa-RTP and P17 were fused to the N-terminal or C-terminal fragment of YFP in the Yn and Yc vectors and infiltrated into N. benthamiana source (A-H) and sink (I-J) leaves in various combinations. The fluorescent signals were visualized by confocal microscopy 2 dpi using the same settings for all observations. (A) Yn-RTP and Yc-RTP; (B) Yn-RTP (Δ5aa) and Yc-RTP (Δ5aa); (C) Yn-RTP and Yc-P17; (D) Yn-P17 and Yc-RTP; (E) Yn-RTP (Δ5aa) and Yc-P17; (F) Yn-P17 and Yc-RTP (Δ5aa). (G) Yn-RTP, Yc-P17 and PLRV clone. (H) Yn-RTP (Δ5aa), Yc-P17 and PLRV clone. (I) Yn-RTP and Yc-P17; (J) Yn-RTP (Δ5aa) and Yc-P17; Bar = 20 μm. Red arrows indicate the nucleus in A and B, the white arrows indicate RTP aggregates in C-J. (TIF) [file ppat.1007451.s005.tif]

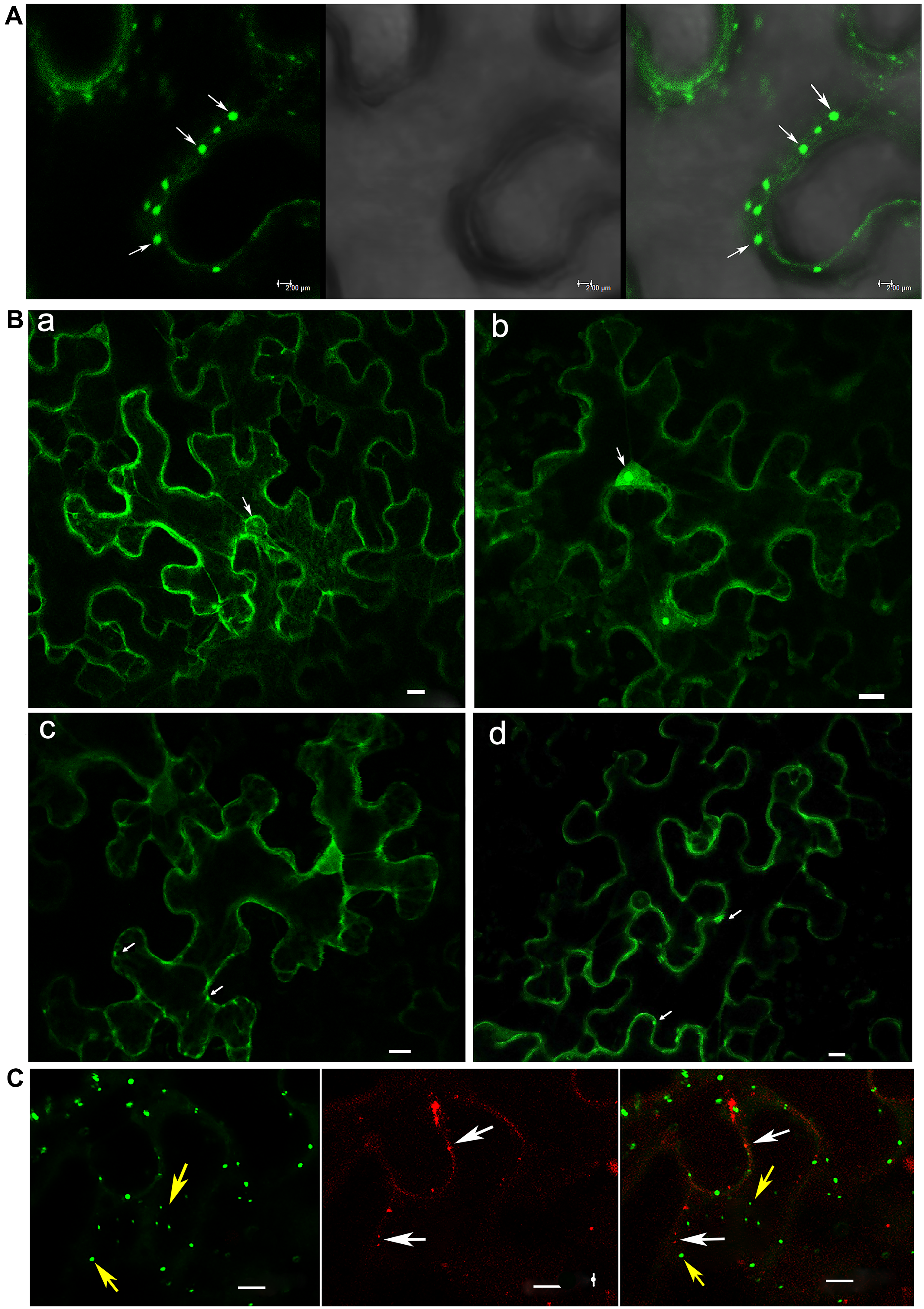

Supplement: S6 Fig — (A) GFP-RTP co-infiltrated with the PLRV infectious clone into source (mature) leaves. Samples were observed with confocal microscopy 3 dpi and the images processed for GFP florescence (left panel), bright-field (middle panel) or merged (right panel). Arrows indicate inclusion–like bodies of varying size. Bar = 2 μm. (B) Full length wild-type RTP and the RTP-Δ5AA fused with GFP (C terminal fusion) infiltrated alone (a-b) or co-infiltrated with the PLRV infectious clone (c-d) into sink (developing) leaves of N. benthamiana. GFP fluorescence was visualized by confocal microscopy 3 dpi. a: GFP-RTP (WT); b: GFP-RTP (Δ5AA); c: GFP-RTP (WT) + PLRV; d: GFP-RTP (Δ5AA) + PLRV. Arrows in a and b indicate the nucleus and arrows in c and d indicate the occasional punctate bodies observed. Bar = 10 μm. (C) GFP-RTP (Δ5AA) and mCherry-PDLP1 co-infiltrated with the PLRV infectious clone into mature N. benthamiana leaves. Left panel, GFP-RTP (Δ5AA) and arrows represent the inclusion bodies in the cytoplasm; Middle, mCherry-PDLP1 and arrows indicate PDLP1-labeled plasmodesmata; Right panel, overlay of left and middle panels. Bar = 10 μm. (TIF) [file ppat.1007451.s006.tif]

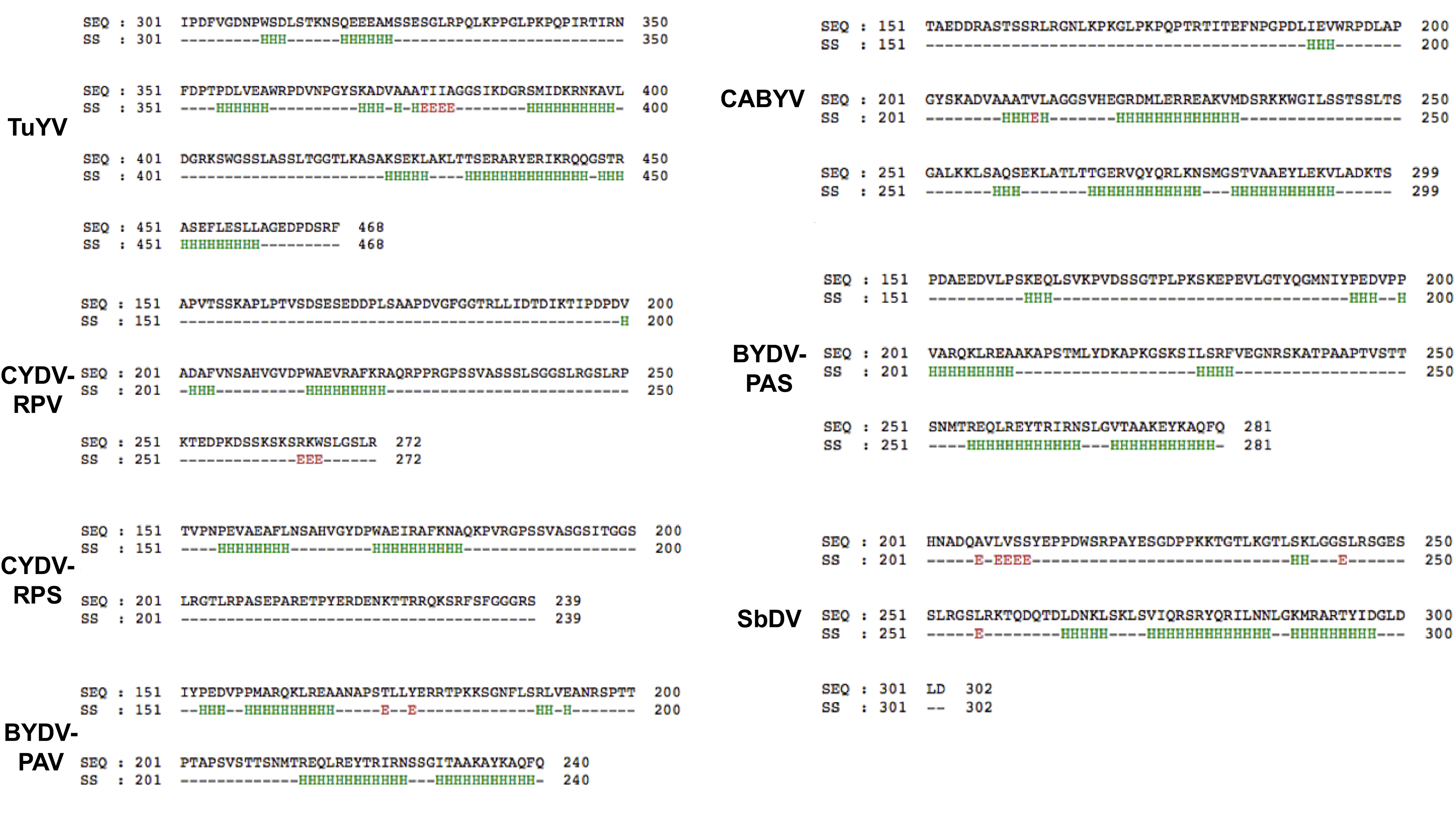

Supplement: S7 Fig — The position and aa acids predicted to form are labeled with an H in green type below the aa designation. SEQ: the position number in the whole RTD domain; SS: secondary structure. (TIF) [file ppat.1007451.s007.tif]

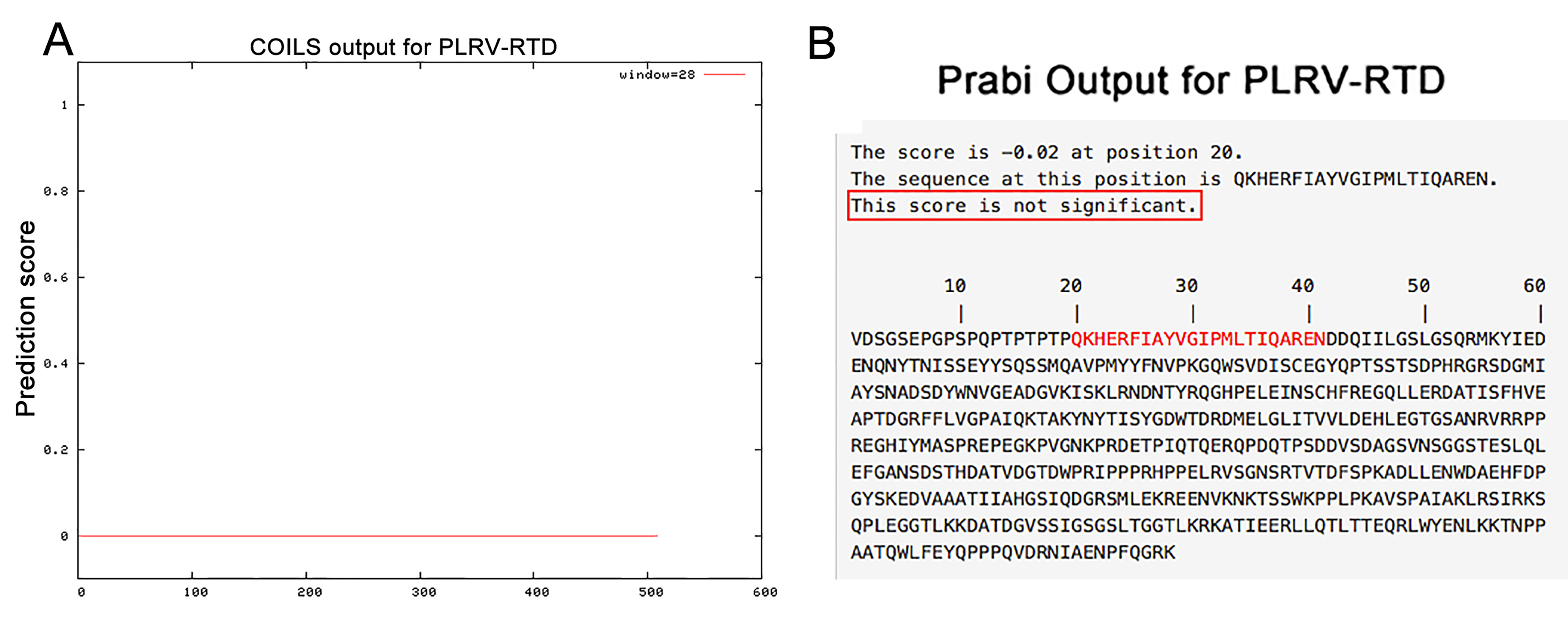

Supplement: S8 Fig — (A) Prediction by COILS algorithm (https://embnet.vital-it.ch/software/COILS_form.html). X-axis represents the position of the amino acid along the RTP. Y-axis: high value represents helix-turn-helix motifs. (B) Prediction by Prabi algorithm (https://npsa-prabi.ibcp.fr/cgi-bin/npsa_automat.pl?page=/NPSA/npsa_hth.html). The red rectangular box showed there is no significant helix-turn-helix motif in PLRV RTD. (TIF) [file ppat.1007451.s008.tif]
