# Supplementary material for: An aromatic amino acid and associated helix in the C-terminus of the potato leafroll virus minor capsid protein regulate systemic infection and symptom expression
Source: PLoS Pathog. 2018 Nov 15;14(11):e1007451. doi: 10.1371/journal.ppat.1007451 (PMC6264904; doi:10.1371/journal.ppat.1007451)
Supplement: S1 Table — (DOCX) [file ppat.1007451.s009.docx]

**S1 Table. Sequence information of WT-PLRV and mutants from constructed plasmids and cloned from infected plants.**

**>WT-PLRV RTD**

GTAGACTCCGGATCAGAGCCTGGTCCAAGCCCACAACCAACACCCACTCCAACTCCCCAGAAGCACGAGAGATTTATTGCTTACGTTGGCATACCTATGCTAACCATTCAGGCCAGGGAGAACGACGACCAAATCATATTGGGTTCCTTAGGGAGCCAAAGGATGAAATATATAGAGGACGAGAACCAGAACTATACAAATATTAGTTCTGAGTATTACTCTCAATCGAGTATGCAAGCCGTCCCTATGTATTATTTCAATGTCCCGAAAGGGCAATGGTCAGTCGATATCAGCTGCGAAGGGTATCAACCCACTAGCAGCACCTCGGATCCACACCGGGGTAGGAGTGACGGGATGATCGCGTATTCAAACGCGGATTCCGATTACTGGAATGTTGGTGAAGCGGATGGTGTCAAAATTTCGAAGCTACGCAACGATAACACCTACCGCCAAGGTCACCCAGAACTTGAAATTAACTCGTGTCATTTTCGCGAGGGCCAACTCCTTGAACGGGACGCTACAATTAGCTTCCACGTTGAAGCGCCTACTGATGGGCGATTCTTTCTCGTTGGTCCCGCTATCCAGAAAACCGCAAAGTATAACTATACTATCTCATACGGTGACTGGACGGACCGAGACATGGAGCTGGGGCTGATCACTGTGGTGCTTGATGAACATTTAGAAGGCACTGGTTCGGCTAACAGAGTGCGGCGGCCCCCACGGGAGGGCCACATCTATATGGCCTCGCCGCGCGAACCGGAAGGAAAACCGGTTGGAAATAAACCAAGGGACGAAACCCCGATACAAACGCAGGAAAGACAACCTGATCAAACTCCGTCTGACGACGTATCCGATGCTGGTTCGGTAAACAGCGGCGGCTCAACTGAGTCGCTGCAATTGGAGTTCGGAGCAAACTCAGATAGTACCCACGATGCTACAGTCGATGGTACAGACTGGCCCAGAATTCCTCCACCAAGGCACCCACCCGAACTTAGAGTTTCCGGCAATTCAAGAACTGTTACTGACTTTTCTCCGAAAGCCGATCTATTGGAGAATTGGGATGCCGAACACTTCGACCCTGGTTATTCCAAAGAAGATGTCGCTGCTGCTACTATTATAGCGCACGGCAGTATTCAAGATGGGCGAAGTATGCTGGAGAAGAGAGAGGAAAATGTCAAGAACAAAACCTCCTCCTGGAAGCCCCCGTTACCTAAAGCGGTGAGCCCAGCCATAGCCAAATTGCGCTCGATTCGCAAATCCCAACCCCTCGAGGGAGGGACCCTTAAGAAAGACGCCACTGATGGTGTCTCATCTATTGGCAGTGGTTCTCTAACAGGTGGCACGCTTAAGAGGAAGGCAACTATTGAAGAGCGTTTACTGCAGACCTTAACAACTGAACAAAGGCTGTGGTACGAGAATTTGAAGAAAACTAACCCTCCAGCTGCCACCCAATGGCTGTTTGAATATCAGCCACCTCCCCAAGTAGATAGAAACATAGCTGAAAATCCATTCCAAGGGAGGAAATGA

**> Mut-∆5670**

GTAGACTCCGGATCAGAGCCTGGTCCAAGCCCACAACCAACACCCACTCCAACTCCCCAGAAGCACGAGAGATTTATTGCTTACGTTGGCATACCTATGCTAACCATTCAGGCCAGGGAGAACGACGACCAAATCATATTGGGTTCCTTAGGGAGCCAAAGGATGAAATATATAGAGGACGAGAACCAGAACTATACAAATATTAGTTCTGAGTATTACTCTCAATCGAGTATGCAAGCCGTCCCTATGTATTATTTCAATGTCCCGAAAGGGCAATGGTCAGTCGATATCAGCTGCGAAGGGTATCAACCCACTAGCAGCACCTCGGATCCACACCGGGGTAGGAGTGACGGGATGATCGCGTATTCAAACGCGGATTCCGATTACTGGAATGTTGGTGAAGCGGATGGTGTCAAAATTTCGAAGCTACGCAACGATAACACCTACCGCCAAGGTCACCCAGAACTTGAAATTAACTCGTGTCATTTTCGCGAGGGCCAACTTCTTGAACGGGACGCTACAATTAGCTTCCACGTTGAAGCGCCTACTGATGGGCGATTCTTTCTCGTTGGTCCCGCTATCCAGAAAACCGCAAAGTATAACTATACTATCTCATACGGTGACTGGACGGACCGAGACATGGAGCTGGGGCTGATCACTGTGGTGCTTGATGAACATTTAGAAGGCACTGGTTCGGCTAACAGAGTGCGGCGGCCCCCACGGGAGGGCCACATCTATATGGCCTCGCCGCGCGAACCGGAAGGAAAACCGGTTGGAAATAAACCAAGGGACGAAACCCCGATACAAACGCAGGAAAGACAACCTGATCAAACTCCGTCTGACGACGTATCCGATGCTGGTTCGGTAAACAGCGGCGGCTCAACTGAGTCGCTGCAATTGGAGTTCGGAGCAAACTCAGATAGTACCCACGATGCTACAGTCGATGGTACAGACTGGCCCAGAATTCCTCCACCAAGGCACCCACCCGAACTTAGAGTTTCCGGCAATTCAAGAACTGTTACTGACTTTTCTCCGAAAGCCGATCTATTGGAGAATTGGGATGCCGAACACTTCGACCCTGGTTATTCCAAAGAAGATGTCGCTGCTGCTACTATTATAGCGCACGGCAGTATTCAAGATGGGCGAAGTATGCTGGAGAAGAGAGAGGAAAATGTCAAGAACAAAACCTCCTCCTGGAAGCCCCCGTTACCTAAAGCGGTGAGCCCAGCCATAGCCAAATTGCGCTCGATTCGCAAATCCCAACCCCTCGAGGGAGGGACCCTTAAGAAAGACGCCACTGATGGTGTCTCATCTATTGGCAGTGGTTCTCTAACAGGTGGCACGCTTAAGAGGAAGGCAACTATTGAAGAGCGTTTACTGCAGACCTTAACAACTGAACAAAGGCTGTGGTACGAGAATTTGAAGAAAACTAACCCTCCAGCTGCCACCCAATGGTGA

**> Mut-∆5685**

GTAGACTCCGGATCAGAGCCTGGTCCAAGCCCACAACCAACACCCACTCCAACTCCCCAGAAGCACGAGAGATTTATTGCTTACGTTGGCATACCTATGCTAACCATTCAGGCCAGGGAGAACGACGACCAAATCATATTGGGTTCCTTAGGGAGCCAAAGGATGAAATATATAGAGGACGAGAACCAGAACTATACAAATATTAGTTCTGAGTATTACTCTCAATCGAGTATGCAAGCCGTCCCTATGTATTATTTCAATGTCCCGAAAGGGCAATGGTCAGTCGATATCAGCTGCGAAGGGTATCAACCCACTAGCAGCACCTCGGATCCACACCGGGGTAGGAGTGACGGGATGATCGCGTATTCAAACGCGGATTCCGATTACTGGAATGTTGGTGAAGCGGATGGTGTCAAAATTTCGAAGCTACGCAACGATAACACCTACCGCCAAGGTCACCCAGAACTTGAAATTAACTCGTGTCATTTTCGCGAGGGCCAACTCCTTGAACGGGACGCTACAATTAGCTTCCACGTTGAAGCGCCTACTGATGGGCGATTCTTTCTCGTTGGTCCCGCTATCCAGAAAACCGCAAAGTATAACTATACTATCTCATACGGTGACTGGACGGACCGAGACATGGAGCTGGGGCTGATCACTGTGGTGCTTGATGAACATTTAGAAGGCACTGGTTCGGCTAACAGAGTGCGGCGGCCCCCACGGGAGGGCCACATCTATATGGCCTCGCCGCGCGAACCGGAAGGAAAACCGGTTGGAAATAAACCAAGGGACGAAACCCCGATACAAACGCAGGAAAGACAACCTGATCAAACTCCGTCTGACGACGTATCCGATGCTGGTTCGGTAAACAGCGGCGGCTCAACTGAGTCGCTGCAATTGGAGTTCGGAGCAAACTCAGATAGTACCCACGATGCTACAGTCGATGGTACAGACTGGCCCAGAATTCCTCCACCAAGGCACCCACCCGAACTTAGAGTTTCCGGCAATTCAAGAACTGTTACTGACTTTTCTCCGAAAGCCGATCTATTGGAGAATTGGGATGCCGAACACTTCGACCCTGGTTATTCCAAAGAAGATGTCGCTGCTGCTACTATTATAGCGCACGGCAGTATTCAAGATGGGCGAAGTATGCTGGAGAAGAGAGAGGAAAATGTCAAGAACAAAACCTCCTCCTGGAAGCCCCCGTTACCTAAAGCGGTGAGCCCAGCCATAGCCAAATTGCGCTCGATTCGCAAATCCCAACCCCTCGAGGGAGGGACCCTTAAGAAAGACGCCACTGATGGTGTCTCATCTATTGGCAGTGGTTCTCTAACAGGTGGCACGCTTAAGAGGAAGGCAACTATTGAAGAGCGTTTACTGCAGACCTTAACAACTGAACAAAGGCTGTGGTACGAGAATTTGAAGAAAACTAACCCTCCAGCTGCCACCCAATGGCTGTTTGAATATCAGTGA

**>Mut-∆5670-5685**

GTAGACTCCGGATCAGAGCCTGGTCCAAGCCCACAACCAACACCCACTCCAACTCCCCAGAAGCACGAGAGATTTATTGCTTACGTTGGCATACCTATGCTAACCATTCAGGCCAGGGAGAACGACGACCAAATCATATTGGGTTCCTTAGGGAGCCAAAGGATGAAATATATAGAGGACGAGAACCAGAACTATACAAATATTAGTTCTGAGTATTACTCTCAATCGAGTATGCAAGCCGTCCCTATGTATTATTTCAATGTCCCGAAAGGGCAATGGTCAGTCGATATCAGCTGCGAAGGGTATCAACCCACTAGCAGCACCTCGGATCCACACCGGGGTAGGAGTGACGGGATGATCGCGTATTCAAACGCGGATTCCGATTACTGGAATGTTGGTGAAGCGGATGGTGTCAAAATTTCGAAGCTACGCAACGATAACACCTACCGCCAAGGTCACCCAGAACTTGAAATTAACTCGTGTCATTTTCGCGAGGGCCAACTCCTTGAACGGGACGCTACAATTAGCTTCCACGTTGAAGCGCCTACTGATGGGCGATTCTTTCTCGTTGGTCCCGCTATCCAGAAAACCGCAAAGTATAACTATACTATCTCATACGGTGACTGGACGGACCGAGACATGGAGCTGGGGCTGATCACTGTGGTGCTTGATGAACATTTAGAAGGCACTGGTTCGGCTAACAGAGTGCGGCGGCCCCCACGGGAGGGCCACATCTATATGGCCTCGCCGCGCGAACCGGAAGGAAAACCGGTTGGAAATAAACCAAGGGACGAAACCCCGATACAAACGCAGGAAAGACAACCTGATCAAACTCCGTCTGACGACGTATCCGATGCTGGTTCGGTAAACAGCGGCGGCTCAACTGAGTCGCTGCAATTGGAGTTCGGAGCAAACTCAGATAGTACCCACGATGCTACAGTCGATGGTACAGACTGGCCCAGAATTCCTCCACCAAGGCACCCACCCGAACTTAGAGTTTCCGGCAATTCAAGAACTGTTACTGACTTTTCTCCGAAAGCCGATCTATTGGAGAATTGGGATGCCGAACACTTCGACCCTGGTTATTCCAAAGAAGATGTCGCTGCTGCTACTATTATAGCGCACGGCAGTATTCAAGATGGGCGAAGTATGCTGGAGAAGAGAGAGGAAAATGTCAAGAACAAAACCTCCTCCTGGAAGCCCCCGTTACCTAAAGCGGTGAGCCCAGCCATAGCCAAATTGCGCTCGATTCGCAAATCCCAACCCCTCGAGGGAGGGACCCTTAAGAAAGACGCCACTGATGGTGTCTCATCTATTGGCAGTGGTTCTCTAACAGGTGGCACGCTTAAGAGGAAGGCAACTATTGAAGAGCGTTTACTGCAGACCTTAACAACTGAACAAAGGCTGTGGTACGAGAATTTGAAGAAAACTAACCCTCCAGCTGCCACCCAATGGCCACCTCCCCAAGTAGATAGAAACATAGCTGAAAATCCATTCCAAGGGAGGAAATGA

**PLRV wild-type virus and mutants in *N. benthamiana,* and *P. floridana* plants at 7 wpi**

**>WT-PLRV RTD** **(7 wpi)**

GTAGACTCCGGATCAGAGCCTGGTCCAAGCCCACAACCAACACCCACTCCAACTCCCCAGAAGCACGAGAGATTTATTGCTTACGTTGGCATACCTATGCTAACCATTCAGGCCAGGGAGAACGACGACCAAATCATATTGGGTTCCTTAGGGAGCCAAAGGATGAAATATATAGAGGACGAGAACCAGAACTATACAAATATTAGTTCTGAGTATTACTCTCAATCGAGTATGCAAGCCGTCCCTATGTATTATTTCAATGTCCCGAAAGGGCAATGGTCAGTCGATATCAGCTGCGAAGGGTATCAACCCACTAGCAGCACCTCGGATCCACACCGGGGTAGGAGTGACGGGATGATCGCGTATTCAAACGCGGATTCCGATTACTGGAATGTTGGTGAAGCGGATGGTGTCAAAATTTCGAAGCTACGCAACGATAACACCTACCGCCAAGGTCACCCAGAACTTGAAATTAACTCGTGTCATTTTCGCGAGGGCCAACTCCTTGAACGGGACGCTACAATTAGCTTCCACGTTGAAGCGCCTACTGATGGGCGATTCTTTCTCGTTGGTCCCGCTATCCAGAAAACCGCAAAGTATAACTATACTATCTCATACGGTGACTGGACGGACCGAGACATGGAGCTGGGGCTGATCACTGTGGTGCTTGATGAACATTTAGAAGGCACTGGTTCGGCTAACAGAGTGCGGCGGCCCCCACGGGAGGGCCACATCTATATGGCCTCGCCGCGCGAACCGGAAGGAAAACCGGTTGGAAATAAACCAAGGGACGAAACCCCGATACAAACGCAGGAAAGACAACCTGATCAAACTCCGTCTGACGACGTATCCGATGCTGGTTCGGTAAACAGCGGCGGCTCAACTGAGTCGCTGCAATTGGAGTTCGGAGCAAACTCAGATAGTACCCACGATGCTACAGTCGATGGTACAGACTGGCCCAGAATTCCTCCACCAAGGCACCCACCCGAACTTAGAGTTTCCGGCAATTCAAGAACTGTTACTGACTTTTCTCCGAAAGCCGATCTATTGGAGAATTGGGATGCCGAACACTTCGACCCTGGTTATTCCAAAGAAGATGTCGCTGCTGCTACTATTATAGCGCACGGCAGTATTCAAGATGGGCGAAGTATGCTGGAGAAGAGAGAGGAAAATGTCAAGAACAAAACCTCCTCCTGGAAGCCCCCGTTACCTAAAGCGGTGAGCCCAGCCATAGCCAAATTGCGCTCGATTCGCAAATCCCAACCCCTCGAGGGAGGGACCCTTAAGAAAGACGCCACTGATGGTGTCTCATCTATTGGCAGTGGTTCTCTAACAGGTGGCACGCTTAAGAGGAAGGCAACTATTGAAGAGCGTTTACTGCAGACCTTAACAACTGAACAAAGGCTGTGGTACGAGAATTTGAAGAAAACTAACCCTCCAGCTGCCACCCAATGGCTGTTTGAATATCAGCCACCTCCCCAAGTAGATAGAAACATAGCTGAAAATCCATTCCAAGGGAGGAAATGA

**> Mut-∆5670 (7 wpi)**

GTAGACTCCGGATCAGAGCCTGGTCCAAGCCCACAACCAACACCCACTCCAACTCCCCAGAAGCACGAGAGATTTATTGCTTACGTTGGCATACCTATGCTAACCATTCAGGCCAGGGAGAACGACGACCAAATCATATTGGGTTCCTTAGGGAGCCAAAGGATGAAATATATAGAGGACGAGAACCAGAACTATACAAATATTAGTTCTGAGTATTACTCTCAATCGAGTATGCAAGCCGTCCCTATGTATTATTTCAATGTCCCGAAAGGGCAATGGTCAGTCGATATCAGCTGCGAAGGGTATCAACCCACTAGCAGCACCTCGGATCCACACCGGGGTAGGAGTGACGGGATGATCGCGTATTCAAACGCGGATTCCGATTACTGGAATGTTGGTGAAGCGGATGGTGTCAAAATTTCGAAGCTACGCAACGATAACACCTACCGCCAAGGTCACCCAGAACTTGAAATTAACTCGTGTCATTTTCGCGAGGGCCAACTCCTTGAACGGGACGCTACAATTAGCTTCCACGTTGAAGCGCCTACTGATGGGCGATTCTTTCTCGTTGGTCCCGCTATCCAGAAAACCGCAAAGTATAACTATACTATCTCATACGGTGACTGGACGGACCGAGACATGGAGCTGGGGCTGATCACTGTGGTGCTTGATGAACATTTAGAAGGCACTGGTTCGGCTAACAGAGTGCGGCGGCCCCCACGGGAGGGCCACATCTATATGGCCTCGCCGCGCGAACCGGAAGGAAAACCGGTTGGAAATAAACCAAGGGACGAAACCCCGATACAAACGCAGGAAAGACAACCTGATCAAACTCCGTCTGACGACGTATCCGATGCTGGTTCGGTAAACAGCGGCGGCTCAACTGAGTCGCTGCAATTGGAGTTCGGAGCAAACTCAGATAGTACCCACGATGCTACAGTCGATGGTACAGACTGGCCCAGAATTCCTCCACCAAGGCACCCACCCGAACTTAGAGTTTCCGGCAATTCAAGAACTGTTACTGACTTTTCTCCGAAAGCCGATCTATTGGAGAATTGGGATGCCGAACACTTCGACCCTGGTTATTCCAAAGAAGATGTCGCTGCTGCTACTATTATAGCGCACGGCAGTATTCAAGATGGGCGAAGTATGCTGGAGAAGAGAGAGGAAAATGTCAAGAACAAAACCTCCTCCTGGAAGCCCCCGTTACCTAAAGCGGTGAGCCCAGCCATAGCCAAATTGCGCTCGATTCGCAAATCCCAACCCCTCGAGGGAGGGACCCTTAAGAAAGACGCCACTGATGGTGTCTCATCTATTGGCAGTGGTTCTCTAACAGGTGGCACGCTTAAGAGGAAGGCAACTATTGAAGAGCGTTTACTGCAGACCTTAACAACTGAACAAAGGCTGTGGTACGAGAATTTGAAGAAAACTAACCCTCCAGCTGCCACCCAATGGTGA

**> Mut-∆5685 (7 wpi)**

GTAGACTCCGGATCAGAGCCTGGTCCAAGCCCACAACCAACACCCACTCCAACTCCCCAGAAGCACGAGAGATTTATTGCTTACGTTGGCATACCTATGCTAACCATTCAGGCCAGGGAGAACGACGACCAAATCATATTGGGTTCCTTAGGGAGCCAAAGGATGAAATATATAGAGGACGAGAACCAGAACTATACAAATATTAGTTCTGAGTATTACTCTCAATCGAGTATGCAAGCCGTCCCTATGTATTATTTCAATGTCCCGAAAGGGCAATGGTCAGTCGATATCAGCTGCGAAGGGTATCAACCCACTAGCAGCACCTCGGATCCACACCGGGGTAGGAGTGACGGGATGATCGCGTATTCAAACGCGGATTCCGATTACTGGAATGTTGGTGAAGCGGATGGTGTCAAAATTTCGAAGCTACGCAACGATAACACCTACCGCCAAGGTCACCCAGAACTTGAAATTAACTCGTGTCATTTTCGCGAGGGCCAACTCCTTGAACGGGACGCTACAATTAGCTTCCACGTTGAAGCGCCTACTGATGGGCGATTCTTTCTCGTTGGTCCCGCTATCCAGAAAACCGCAAAGTATAACTATACTATCTCATACGGTGACTGGACGGACCGAGACATGGAGCTGGGGCTGATCACTGTGGTGCTTGATGAACATTTAGAAGGCACTGGTTCGGCTAACAGAGTGCGGCGGCCCCCACGGGAGGGCCACATCTATATGGCCTCGCCGCGCGAACCGGAAGGAAAACCGGTTGGAAATAAACCAAGGGACGAAACCCCGATACAAACGCAGGAAAGACAACCTGATCAAACTCCGTCTGACGACGTATCCGATGCTGGTTCGGTAAACAGCGGCGGCTCAACTGAGTCGCTGCAATTGGAGTTCGGAGCAAACTCAGATAGTACCCACGATGCTACAGTCGATGGTACAGACTGGCCCAGAATTCCTCCACCAAGGCACCCACCCGAACTTAGAGTTTCCGGCAATTCAAGAACTGTTACTGACTTTTCTCCGAAAGCCGATCTATTGGAGAATTGGGATGCCGAACACTTCGACCCTGGTTATTCCAAAGAAGATGTCGCTGCTGCTACTATTATAGCGCACGGCAGTATTCAAGATGGGCGAAGTATGCTGGAGAAGAGAGAGGAAAATGTCAAGAACAAAACCTCCTCCTGGAAGCCCCCGTTACCTAAAGCGGTGAGCCCAGCCATAGCCAAATTGCGCTCGATTCGCAAATCCCAACCCCTCGAGGGAGGGACCCTTAAGAAAGACGCCACTGATGGTGTCTCATCTATTGGCAGTGGTTCTCTAACAGGTGGCACGCTTAAGAGGAAGGCAACTATTGAAGAGCGTTTACTGCAGACCTTAACAACTGAACAAAGGCTGTGGTACGAGAATTTGAAGAAAACTAACCCTCCAGCTGCCACCCAATGGCTGTTTGAATATCAGTGA

**>Mut-∆5670-5685 (7 wpi)**

GTAGACTCCGGATCAGAGCCTGGTCCAAGCCCACAACCAACACCCACTCCAACTCCCCAGAAGCACGAGAGATTTATTGCTTACGTTGGCATACCTATGCTAACCATTCAGGCCAGGGAGAACGACGACCAAATCATATTGGGTTCCTTAGGGAGCCAAAGGATGAAATATATAGAGGACGAGAACCAGAACTATACAAATATTAGTTCTGAGTATTACTCTCAATCGAGTATGCAAGCCGTCCCTATGTATTATTTCAATGTCCCGAAAGGGCAATGGTCAGTCGATATCAGCTGCGAAGGGTATCAACCCACTAGCAGCACCTCGGATCCACACCGGGGTAGGAGTGACGGGATGATCGCGTATTCAAACGCGGATTCCGATTACTGGAATGTTGGTGAAGCGGATGGTGTCAAAATTTCGAAGCTACGCAACGATAACACCTACCGCCAAGGTCACCCAGAACTTGAAATTAACTCGTGTCATTTTCGCGAGGGCCAACTCCTTGAACGGGACGCTACAATTAGCTTCCACGTTGAAGCGCCTACTGATGGGCGATTCTTTCTCGTTGGTCCCGCTATCCAGAAAACCGCAAAGTATAACTATACTATCTCATACGGTGACTGGACGGACCGAGACATGGAGCTGGGGCTGATCACTGTGGTGCTTGATGAACATTTAGAAGGCACTGGTTCGGCTAACAGAGTGCGGCGGCCCCCACGGGAGGGCCACATCTATATGGCCTCGCCGCGCGAACCGGAAGGAAAACCGGTTGGAAATAAACCAAGGGACGAAACCCCGATACAAACGCAGGAAAGACAACCTGATCAAACTCCGTCTGACGACGTATCCGATGCTGGTTCGGTAAACAGCGGCGGCTCAACTGAGTCGCTGCAATTGGAGTTCGGAGCAAACTCAGATAGTACCCACGATGCTACAGTCGATGGTACAGACTGGCCCAGAATTCCTCCACCAAGGCACCCACCCGAACTTAGAGTTTCCGGCAATTCAAGAACTGTTACTGACTTTTCTCCGAAAGCCGATCTATTGGAGAATTGGGATGCCGAACACTTCGACCCTGGTTATTCCAAAGAAGATGTCGCTGCTGCTACTATTATAGCGCACGGCAGTATTCAAGATGGGCGAAGTATGCTGGAGAAGAGAGAGGAAAATGTCAAGAACAAAACCTCCTCCTGGAAGCCCCCGTTACCTAAAGCGGTGAGCCCAGCCATAGCCAAATTGCGCTCGATTCGCAAATCCCAACCCCTCGAGGGAGGGACCCTTAAGAAAGACGCCACTGATGGTGTCTCATCTATTGGCAGTGGTTCTCTAACAGGTGGCACGCTTAAGAGGAAGGCAACTATTGAAGAGCGTTTACTGCAGACCTTAACAACTGAACAAAGGCTGTGGTACGAGAATTTGAAGAAAACTAACCCTCCAGCTGCCACCCAATGGCCACCTCCCCAAGTAGATAGAAACATAGCTGAAAATCCATTCCAAGGGAGGAAATGA

**>Rev**

GTAGACTCCGGATCAGAGCCTGGTCCAAGCCCACAACCAACACCCACTCCAACTCCCCAGAAGCACGAGAGATTTATTGCTTACGTTGGCATACCTATGCTAACCATTCAGGCCAGGGAGAACGACGACCAAATCATATTGGGTTCCTTAGGGAGCCAAAGGATGAAATATATAGAGGACGAGAACCAGAACTATACAAATATTAGTTCTGAGTATTACTCTCAATCGAGTATGCAAGCCGTCCCTATGTATTATTTCAATGTCCCGAAAGGGCAATGGTCAGTCGATATCAGCTGCGAAGGGTATCAACCCACTAGCAGCACCTCGGATCCACACCGGGGTAGGAGTGACGGGATGATCGCGTATTCAAACGCGGATTCCGATTACTGGAATGTTGGTGAAGCGGATGGTGTCAAAATTTCGAAGCTACGCAACGATAACACCTACCGCCAAGGTCACCCAGAACTTGAAATTAACTCGTGTCATTTTCGCGAGGGCCAACTCCTTGAACGGGACGCTACAATTAGCTTCCACGTTGAAGCGCCTACTGATGGGCGATTCTTTCTCGTTGGTCCCGCTATCCAGAAAACCGCAAAGTATAACTATACTATCTCATACGGTGACTGGACGGACCGAGACATGGAGCTGGGGCTGATCACTGTGGTGCTTGATGAACATTTAGAAGGCACTGGTTCGGCTAACAGAGTGCGGCGGCCCCCACGGGAGGGCCACATCTATATGGCCTCGCCGCGCGAACCGGAAGGAAAACCGGTTGGAAATAAACCAAGGGACGAAACCCCGATACAAACGCAGGAAAGACAACCTGATCAAACTCCGTCTGACGACGTATCCGATGCTGGTTCGGTAAACAGCGGCGGCTCAACTGAGTCGCTGCAATTGGAGTTCGGAGCAAACTCAGATAGTACCCACGATGCTACAGTCGATGGTACAGACTGGCCCAGAATTCCTCCACCAAGGCACCCACCCGAACTTAGAGTTTCCGGCAATTCAAGAACTGTTACTGACTTTTCTCCGAAAGCCGATCTATTGGAGAATTGGGATGCCGAACACTTCGACCCTGGTTATTCCAAAGAAGATGTCGCTGCTGCTACTATTATAGCGCACGGCAGTATTCAAGATGGGCGAAGTATGCTGGAGAAGAGAGAGGAAAATGTCAAGAACAAAACCTCCTCCTGGAAGCCCCCGTTACCTAAAGCGGTGAGCCCAGCCATAGCCAAATTGCGCTCGATTCGCAAATCCCAACCCCTCGAGGGAGGGACCCTTAAGAAAGACGCCACTGATGGTGTCTCATCTATTGGCAGTGGTTCTCTAACAGGTGGCACGCTTAAGAGGAAGGCAACTATTGAAGAGCGTTTACTGCAGACCTTAACAACTGAACAAAGGCTGTGGTACGAGAATTTGAAGAAAACTAACCCTCCAGCTGCCACCCAATGGTGGTTTACTGCAGACCTTAACAACTGAACAAAGGCTGTGGTACGAGAATTTGAAGAAAACTAACCCTCCAGCTGCCACCCAATGGTGA

**>Mut-LYE (5 wpi)**

…GCGCTCGATTCGCAAATCCCAACCCCTCGAGGGAGGGACCCTTAAGAAAGACGCCACTGATGGTGTCTCATCTATTGGCAGTGGTTCTCTAACAGGTGGCACGCTTAAGAGGAAGGCAACTATTGAAGAGCGTTTACTGCAGACCTTAACAACTGAACAAAGGCTGTGGTACGAGAATTTGAAGAAAACTAACCCTCCAGCTGCCACCCAATGGCTGTATGAATATCAGCCACCTCCCCAAGTAGATAGAAACATAGCTGAAAATCCATTCCAAGGGAGGAAATGA

**>Mut-LHE (5 wpi)**

…GCGCTCGATTCGCAAATCCCAACCCCTCGAGGGAGGGACCCTTAAGAAAGACGCCACTGATGGTGTCTCATCTATTGGCAGTGGTTCTCTAACAGGTGGCACGCTTAAGAGGAAGGCAACTATTGAAGAGCGTTTACTGCAGACCTTAACAACTGAACAAAGGCTGTGGTACGAGAATTTGAAGAAAACTAACCCTCCAGCTGCCACCCAATGGCTGCATGAATATCAGCCACCTCCCCAAGTAGATAGAAACATAGCTGAAAATCCATTCCAAGGGAGGAAATGA

**>Mut-LFK (5 wpi)**

…CAAGAACAAAACCTCCTCCTGGAAGCCCCCGTTACCTAAAGCGGTGAGCCCAGCCATAGCCAAATTGCGCTCGATTCGCAAATCCCAACCCCTCGAGGGAGGGACCCTTAAGAAAGACGCCACTGATGGTGTCTCATCTATTGGCAGTGGTTCTCTAACAGGTGGCACGCTTAAGAGGAAGGCAACTATTGAAGAGCGTTTACTGCAGACCTTAACAACTGAACAAAGGCTGTGGTACGAGAATTTGAAGAAAACTAACCCTCCAGCTGCCACCCAATGGCTGTTTAAATATCAGCCACCTCCCCAAGTAGATAGAAACATAGCTGAAAATCCATTCCAAGGGAGGAAATGA

**>Mut-MFE (5 wpi)**

…GCGCTCGATTCGCAAATCCCAACCCCTCGAGGGAGGGACCCTTAAGAAAGACGCCACTGATGGTGTCTCATCTATTGGCAGTGGTTCTCTAACAGGTGGCACGCTTAAGAGGAAGGCAACTATTGAAGAGCGTTTACTGCAGACCTTAACAACTGAACAAAGGCTGTGGTACGAGAATTTGAAGAAAACTAACCCTCCAGCTGCCACCCAATGGATGTTTGAATATCAGCCACCTCCCCAAGTAGATAGAAACATAGCTGAAAATCCATTCCAAGGGAGGAAATGA

**>Mut-PFG (5 wpi)**

…GCGCTCGATTCGCAAATCCCAACCCCTCGAGGGAGGGACCCTTAAGAAAGACGCCACTGATGGTGTCTCATCTATTGGCAGTGGTTCTCTAACAGGTGGCACGCTTAAGAGGAAGGCAACTATTGAAGAGCGTTTACTGCAGACCTTAACAACTGAACAAAGGCTGTGGTACGAGAATTTGAAGAAAACTAACCCTCCAGCTGCCACCCAATGGCCGTTTGGATATCAGCCACCTCCCCAAGTAGATAGAAACATAGCTGAAAATCCATTCCAAGGGAGGAAATGA

**>Mut-GFP (5 wpi)**

…AATTGGAGTTCGGAGCAAACTCAGATAGTACCCACGATGCTACAGTCGATGGTACAGACTGGCCCAGAATTCCTCCACCAAGGCACCCACCCGAACTTAGAGTTTCCGGCAATTCAAGAACTGTTACTGACTTTTCTCCGAAAGCCGATCTATTGGAGAATTGGGATGCCGAACACTTCGACCCTGGTTATTCCAAAGAAGATGTCGCTGCTGCTACTATTATAGCGCACGGCAGTATTCAAGATGGGCGAAGTATGCTGGAGAAGAGAGAGGAAAATGTCAAGAACAAAACCTCCTCCTGGAAGCCCCCGTTACCTAAAGCGGTGAGCCCAGCCATAGCCAAATTGCGCTCGATTCGCAAATCCCAACCCCTCGAGGGAGGGACCCTTAAGAAAGACGCCACTGATGGTGTCTCATCTATTGGCAGTGGTTCTCTAACAGGTGGCACGCTTAAGAGGAAGGCAACTATTGAAGAGCGTTTACTGCAGACCTTAACAACTGAACAAAGGCTGTGGTACGAGAATTTGAAGAAAACTAACCCTCCAGCTGCCACCCAATGGGGGTTTCCATATCAGCCACCTCCCCAAGTAGATAGAAACATAGCTGAAAATCCATTCCAAGGGAGGAAATGA

**>Mut-LAE (5 wpi)**

…ATCAAACTCCGTCTGACGACGTATCCGATGCTGGTTCGGTAAACAGCGGCGGCTCAACTGAGTCGCTGCAATTGGAGTTCGGAGCAAACTCAGATAGTACCCACGATGCTACAGTCGATGGTACAGACTGGCCCAGAATTCCTCCACCAAGGCACCCACCCGAACTTAGAGTTTCCGGCAATTCAAGAACTGTTACTGACTTTTCTCCGAAAGCCGATCTATTGGAGAATTGGGATGCCGAACACTTCGACCCTGGTTATTCCAAAGAAGATGTCGCTGCTGCTACTATTATAGCGCACGGCAGTATTCAAGATGGGCGAAGTATGCTGGAGAAGAGAGAGGAAAATGTCAAGAACAAAACCTCCTCCTGGAAGCCCCCGTTACCTAAAGCGGTGAGCCCAGCCATAGCCAAATTGCGCTCGATTCGCAAATCCCAACCCCTCGAGGGAGGGACCCTTAAGAAAGACGCCACTGATGGTGTCTCATCTATTGGCAGTGGTTCTCTAACAGGTGGCACGCTTAAGAGGAAGGCAACTATTGAAGAGCGTTTACTGCAGACCTTAACAACTGAACAAAGGCTGTGGTACGAGAATTTGAAGAAAACTAACCCTCCAGCTGCCACCCAATGGCTGGCTGAATATCAGCCACCTCCCCAAGTAGATAGAAACATAGCTGAAAATCCATTCCAAGGGAGGAAATGA

**>Mut-LEE (5 wpi)**

…ATCAAACTCCGTCTGACGACGTATCCGATGCTGGTTCGGTAAACAGCGGCGGCTCAACTGAGTCGCTGCAATTGGAGTTCGGAGCAAACTCAGATAGTACCCACGATGCTACAGTCGATGGTACAGACTGGCCCAGAATTCCTCCACCAAGGCACCCACCCGAACTTAGAGTTTCCGGCAATTCAAGAACTGTTACTGACTTTTCTCCGAAAGCCGATCTATTGGAGAATTGGGATGCCGAACACTTCGACCCTGGTTATTCCAAAGAAGATGTCGCTGCTGCTACTATTATAGCGCACGGCAGTATTCAAGATGGGCGAAGTATGCTGGAGAAGAGAGAGGAAAATGTCAAGAACAAAACCTCCTCCTGGAAGCCCCCGTTACCTAAAGCGGTGAGCCCAGCCATAGCCAAATTGCGCTCGATTCGCAAATCCCAACCCCTCGAGGGAGGGACCCTTAAGAAAGACGCCACTGATGGTGTCTCATCTATTGGCAGTGGTTCTCTAACAGGTGGCACGCTTAAGAGGAAGGCAACTATTGAAGAGCGTTTACTGCAGACCTTAACAACTGAACAAAGGCTGTGGTACGAGAATTTGAAGAAAACTAACCCTCCAGCTGCCACCCAATGGCTGGAGGAATATCAGCCACCTCCCCAAGTAGATAGAAACATAGCTGAAAATCCATTCCAAGGGAGGAAATGA

**>Mut-AAA (5 wpi)**

…ATCAAACTCCGTCTGACGACGTATCCGATGCTGGTTCGGTAAACAGCGGCGGCTCAACTGAGTCGCTGCAATTGGAGTTCGGAGCAAACTCAGATAGTACCCACGATGCTACAGTCGATGGTACAGACTGGCCCAGAATTCCTCCACCAAGGCACCCACCCGAACTTAGAGTTTCCGGCAATTCAAGAACTGTTACTGACTTTTCTCCGAAAGCCGATCTATTGGAGAATTGGGATGCCGAACACTTCGACCCTGGTTATTCCAAAGAAGATGTCGCTGCTGCTACTATTATAGCGCACGGCAGTATTCAAGATGGGCGAAGTATGCTGGAGAAGAGAGAGGAAAATGTCAAGAACAAAACCTCCTCCTGGAAGCCCCCGTTACCTAAAGCGGTGAGCCCAGCCATAGCCAAATTGCGCTCGATTCGCAAATCCCAACCCCTCGAGGGAGGGACCCTTAAGAAAGACGCCACTGATGGTGTCTCATCTATTGGCAGTGGTTCTCTAACAGGTGGCACGCTTAAGAGGAAGGCAACTATTGAAGAGCGTTTACTGCAGACCTTAACAACTGAACAAAGGCTGTGGTACGAGAATTTGAAGAAAACTAACCCTCCAGCTGCCACCCAATGGGCGGCTGCATATCAGCCACCTCCCCAAGTAGATAGAAACATAGCTGAAAATCCATTCCAAGGGAGGAAATGA

**>Mut-PGE (5 wpi)**

…ATCAAACTCCGTCTGACGACGTATCCGATGCTGGTTCGGTAAACAGCGGCGGCTCAACTGAGTCGCTGCAATTGGAGTTCGGAGCAAACTCAGATAGTACCCACGATGCTACAGTCGATGGTACAGACTGGCCCAGAATTCCTCCACCAAGGCACCCACCCGAACTTAGAGTTTCCGGCAATTCAAGAACTGTTACTGACTTTTCTCCGAAAGCCGATCTATTGGAGAATTGGGATGCCGAACACTTCGACCCTGGTTATTCCAAAGAAGATGTCGCTGCTGCTACTATTATAGCGCACGGCAGTATTCAAGATGGGCGAAGTATGCTGGAGAAGAGAGAGGAAAATGTCAAGAACAAAACCTCCTCCTGGAAGCCCCCGTTACCTAAAGCGGTGAGCCCAGCCATAGCCAAATTGCGCTCGATTCGCAAATCCCAACCCCTCGAGGGAGGGACCCTTAAGAAAGACGCCACTGATGGTGTCTCATCTATTGGCAGTGGTTCTCTAACAGGTGGCACGCTTAAGAGGAAGGCAACTATTGAAGAGCGTTTACTGCAGACCTTAACAACTGAACAAAGGCTGTGGTACGAGAATTTGAAGAAAACTAACCCTCCAGCTGCCACCCAATGGCCGGGTGAATATCAGCCACCTCCCCAAGTAGATAGAAACATAGCTGAAAATCCATTCCAAGGGAGGAAATGA

**>Mut-LPG (5 wpi)**

…ATCAAACTCCGTCTGACGACGTATCCGATGCTGGTTCGGTAAACAGCGGCGGCTCAACTGAGTCGCTGCAATTGGAGTTCGGAGCAAACTCAGATAGTACCCACGATGCTACAGTCGATGGTACAGACTGGCCCAGAATTCCTCCACCAAGGCACCCACCCGAACTTAGAGTTTCCGGCAATTCAAGAACTGTTACTGACTTTTCTCCGAAAGCCGATCTATTGGAGAATTGGGATGCCGAACACTTCGACCCTGGTTATTCCAAAGAAGATGTCGCTGCTGCTACTATTATAGCGCACGGCAGTATTCAAGATGGGCGAAGTATGCTGGAGAAGAGAGAGGAAAATGTCAAGAACAAAACCTCCTCCTGGAAGCCCCCGTTACCTAAAGCGGTGAGCCCAGCCATAGCCAAATTGCGCTCGATTCGCAAATCCCAACCCCTCGAGGGAGGGACCCTTAAGAAAGACGCCACTGATGGTGTCTCATCTATTGGCAGTGGTTCTCTAACAGGTGGCACGCTTAAGAGGAAGGCAACTATTGAAGAGCGTTTACTGCAGACCTTAACAACTGAACAAAGGCTGTGGTACGAGAATTTGAAGAAAACTAACCCTCCAGCTGCCACCCAATGGCTGGGTGGATATCAGCCACCTCCCCAAGTAGATAGAAACATAGCTGAAAATCCATTCCAAGGGAGGAAATGA

**>Mut-PGG (5 wpi)**

…ATCAAACTCCGTCTGACGACGTATCCGATGCTGGTTCGGTAAACAGCGGCGGCTCAACTGAGTCGCTGCAATTGGAGTTCGGAGCAAACTCAGATAGTACCCACGATGCTACAGTCGATGGTACAGACTGGCCCAGAATTCCTCCACCAAGGCACCCACCCGAACTTAGAGTTTCCGGCAATTCAAGAACTGTTACTGACTTTTCTCCGAAAGCCGATCTATTGGAGAATTGGGATGCCGAACACTTCGACCCTGGTTATTCCAAAGAAGATGTCGCTGCTGCTACTATTATAGCGCACGGCAGTATTCAAGATGGGCGAAGTATGCTGGAGAAGAGAGAGGAAAATGTCAAGAACAAAACCTCCTCCTGGAAGCCCCCGTTACCTAAAGCGGTGAGCCCAGCCATAGCCAAATTGCGCTCGATTCGCAAATCCCAACCCCTCGAGGGAGGGACCCTTAAGAAAGACGCCACTGATGGTGTCTCATCTATTGGCAGTGGTTCTCTAACAGGTGGCACGCTTAAGAGGAAGGCAACTATTGAAGAGCGTTTACTGCAGACCTTAACAACTGAACAAAGGCTGTGGTACGAGAATTTGAAGAAAACTAACCCTCCAGCTGCCACCCAATGGCCGGGTGGATATCAGCCACCTCCCCAAGTAGATAGAAACATAGCTGAAAATCCATTCCAAGGGAGGAAATGA

**>NoORF7 RTD (5 wpi, colony 1)**

GTAGACTCCGGATCAGAGCCTGGTCCAAGCCCACAACCAACACCCACTCCAACTCCCCAGAAGCACGAGAGATTTATTGCTTACGTTGGCATACCTATGCTAACCATTCAGGCCAGGGAGAACGACGACCAAATCATATTGGGTTCCTTAGGGAGCCAAAGGATGAAATATATAGAGGACGAGAACCAGAACTATACAAATATTAGTTCTGAGTATTACTCTCAATCGAGTATGCAAGCCGTCCCTATGTATTATTTCAATGTCCCGAAAGGGCAATGGTCAGTCGATATCAGCTGCGAAGGGTATCAACCCACTAGCAGCACCTCGGATCCACACCGGGGTAGGAGTGACGGGATGATCGCGTATTCAAACGCGGATTCCGATTACTGGAATGTTGGTGAAGCGGATGGTGTCAAAATTTCGAAGCTACGCAACGATAACACCTACCGCCAAGGTCACCCAGAACTTGAAATTAACTCGTGTCATTTTCGCGAGGGCCAACTTCTTGAACGGGACGCTACAATTAGCTTCCACGTTGAAGCGCCTACTGATGGGCGATTCTTTCTCGTTGGTCCCGCTATCCAGAAAACCGCAAAGTATAACTATACTATCTCATACGGTGACTGGACGGACCGAGACATGGAGCTGGGGCTGATCACTGTGGTGCTTGATGAACATTTAGAAGGCACTGGTTCGGCTAACAGAGTGCGGCGGCCCCCACGGGAGGGCCACATCTATATGGCCTCGCCGCGCGAACCGGAAGGAAAACCGGTTGGAAATAAACCAAGGGACGAAACCCCGATACAAACGCAGGAAAGACAACCTGATCAAACTCCGTCTGACGACGTATCCGATGCTGGTTCGGTAAACAGCGGCGGCTCAACTGAGTCGCTGCAATTGGAGTTCGGAGCAAACTCAGATAGTACCCACGATGCTACAGTCGATGGTACAGACTGGCCCAGAATTCCTCCACCAAGGCACCCACCCGAACTTAGAGTTTCCGGCAATTCAAGAACTGTTACTGACTTTTCTCCGAAAGCCGATCTATTGGAGAATTGGGATGCCGAACACTTCGACCCTGGTTATTCCAAAGAAGATGTCGCTGCTGCTACTATTATAGCGCACGGCAGTATTCAAGATGGGCGAAGTTTACTGGAGAAGAGAGAGGAAAATGTCAAGAACAAAACCTCCTCCTGGAAGCCCCCGTTACCTAAAGCGGTGAGCCCAGCCATAGCCAAATTGCGCTCGATTCGCAAATCCCAACCCCTCGAGGGAGGGACCCTTAAGAAAGACGCCACTGATGGTGTCTCATCTATTGGCAGTGGTTCTCTAACAGGTGGCACGCTTAAGAGGAAGGCAACTATTGAAGAGCGTTTACTGCAGACCTTAACAACTGAACAAAGGCTGTGGTACGAGAATTTGAAGAAAACTAACCCTCCAGCTGCCACCCAATGGCTGTTTGAATATCAGCCACCTCCCCAAGTAGATAGAAACATAGCTGAAAATCCATTCCAAGGGAGGAAATGA

**>NoORF7 RTD (5 wpi, colony 2)**

GTAGACTCCGGATCAGAGCCTGGTCCAAGCCCACAACCAACACCCACTCCAACTCCCCAGAAGCACGAGAGATTTATTGCTTATGTTGGCATACCTATGCTAACCATTCAGGCCAGGGAGAACGACGACCAAATCATATTGGGTTCCTTAGGGAGCCAAAGGATGAAATATATAGAGGACGAGAACCAGAACTATACAAATATTAGTTCTGAGTATTACTCTCAATCGAGTATGCAAGCCGTCCCTATGTATTATTTCAATGTCCCGAAAGGGCAATGGTCAGTCGATATCAGCTGCGAAGGGTATCAACCCACTAGCAGCACCTCGGATCCACACCGGGGTAGGAGTGACGGGATGATCGCGTATTCAAACGCGGATTCCGATTACTGGAATGTTGGTGAAGCGGATGGTGTCAAAATTTCGAAGCTACGCAACGATAACACCTACCGCCAAGGTCACCCAGAACTTGAAATAAACTCGTGTCATTTTCGCGAGGGCCAACTCCTTGAACGGGACGCTACAATTAGCTTCCACGTTGAAGCGCCTACTGATGGGCGATTCTTTCTCGTTGGTCCCGCTATCCAGAAAACCGCAAAGTATAACTATACTATCTCATACGGTGACTGGACGGACCGAGACATGGAGCTGGGGCTGATCACTGTGGTGCTTGATGAACATTTAGAAGGCACTGGTTCGGCTAACAGAGTGCGGCGGCCCCCACGGGAGGGCCACATCTATATGGCCTCGCCGCGCGAACCGGAAGGAAAACCGGTTGGAAATAAACCAAGGGACGAAACCCCGATACAAACGCAGGAAAGACAACCTGATCAAACTCCGTCTGACGACGTATCCGATGCTGGTTCGGTAAACAGCGGCGGCTCAACTGAGTCGCTGCAATTGGAGTTCGGAGCAAACTCAGATAGTACCCACGATGCTACAGTCGATGGTACAGACTGGCCCAGAATTCCTCCACCAAGGCACCCACCCGAACTTAGAGTTTCCGGCAATTCAAGAACTGTTACTGACTTTTCTCCGAAAGCCGATCTATTGGAGAATTGGGATGCCGAACACTTCGACCCTGGTTATTCCAAAGAAGATGTCGCTGCTGCTACTATTATAGCGCACGGCAGTATTCAAGATGGGCGAAGTTTACTGGAGAAGAGAGAGGAAAATGTCAAGAACAAAACCTCCTCCTGGAAGCCCCCGTTACCTAAAGCGGTGAGCCCAGCCATAGCCAAATTGCGCTCGATTCGCAAATCCCAACCCCTCGAGGGAGGGACCCTTAAGAAAGACGCCACTGATGGTGTCTCATCTATTGGCAGTGGTTCTCTAACAGGTGGCACGCTTAAGAGGAAGGCAACTATTGAAGAGCGTTTACTGCAGACCTTAACAACTGAACAAAGGCTGTGGTACGAGAATTTGAAGAAAACTAACCCTCCAGCTGCCACCCAATGGCTGTTTGAATATCAGCCACCTCCCCAAGTAGATAGAAACATAGCTGAAAATCCATTCCAAGGGAGGAAATGA

**>NoORF7 RTD (5 wpi, colony 3)**

GTAGACTCCGGATCAGAGCCTGGTCCAAGCCCACAACCAACACCCACTCCAACTCCCCAGAAGCACGAGAGCTTTATTGCTTACGTTGGCATACCTATGCTAACCATTCAGGCCAGGGAGAACGACGACCAAATCATATTGGGTTCCTTAGGGAGCCAAAGGATGAAATATATAGAGGACGAGAACCAGAACTATACAAATATTAGTTCTGAGTATTACTCTCAATCGAGTATGCAAGCCGTCCCTATGTATTATTTCAATGTCCCGAAAGGGCAATGGTCAGTCGATATCAGCTGCGAAGGGTATCAACCCACTAGCAGCACCTCGGATCCACACCGGGGTAGGAGTGACGGGATGATCGCGTATTCAAACGCGGATTCCGATTACTGGAATGTTGGTGAAGCGGATGGTGTCAAAATTTCGAAGCTACGCAACGATAACACCTACCGCCAAGGTCACCCAGAACTTGAAATTAACTCGTGTCATTTTCGCGAGGGCCAACTCCTTGAACGGGACGCTACAATTAGCTTCCACGTTGAAGCGCCTACTGATGGGCGATTCTTTCTCGTTGGTCCCGCTATCCAGAAAACCGCAAAGTATAACTATACTATCTCATACGGTGACTGGACGGACCGAGACATGGAGCTGGGGCTGATCACTGTGGTGCTTGATGAACATTTAGAAGGCACTGGTTCGGCTAACAGAGTGCGGCGGCCCCCACGGGAGGGCCACATCTATATGGCCTCGCCGCGCGAACCGGAAGGAAAACCGGTTGGAAATAAACCAAGGGACGAAACCCCGATACAAACGCAGGAAAGACAACCTGATCAAACTCCGTCTGACGACGTATCCGATGCTGGTTCGGTAAACAGCGGCGGCTCAACTGAGTCGCTGCAATTGGAGTTCGGAGCAAACTCAGATAGTACCCACGATGCTACAGTCGATGGTACAGACTGGCCCAGAATTCCTCCACCAAGGCACCCACCCGAACTTAGAGTTTCCGGCAATTCAAGAACTGTTACTGACTTTTCTCCGAAAGCCGATCTATTGGAGAATTGGGATGCCGAACACTTCGACCCTGGTTATTCCAAAGAAGATGTCGCTGCTGCTACTATTATAGCGCACGGCAGTATTCAAGATGGGCGAAGTTTACTGGAGAAGAGAGAGGAAAATGTCAAGAACAAAACCTCCTCCTGGAAGCCCCCGTTACCTAAAGCGGTGAGCCCAGCCATAGCCAAATTGCGCTCGATTCGCAAATCCCAACCCCTCGAGGGAGGGACCCTTAAGAAAGACGCCACTGATGGTGTCTCATCTATTGGCAGTGGTTCTCTAACAGGTGGCACGCTTAAGAGGAAGGCAACTATTGAAGAGCGTTTACTGCAGACCTTAACAACTGAACAAAGGCTGTGGTACGAGAATTTGAAGAAAACTAACCCTCCAGCTGCCACCCAATGGCTGTTTGAATATCAGCCACCTCCCCAAGTAGATAGAAACATAGCTGAAAATCCATTCCAAGGGAGGAAATGA
