# Supplementary material for: An aromatic amino acid and associated helix in the C-terminus of the potato leafroll virus minor capsid protein regulate systemic infection and symptom expression
Source: PLoS Pathog. 2018 Nov 15;14(11):e1007451. doi: 10.1371/journal.ppat.1007451 (PMC6264904; doi:10.1371/journal.ppat.1007451)
Supplement: S2 Table — (DOCX) [file ppat.1007451.s010.docx]

**S2 Table. Pathogenicity and aphid transmissibility of PLRV revertants infecting hairy nightshade plants.**

| **PLRV/mutants** | **No. plants infected/No. plants agroinfiltrated** | **No. plants with symptom** | **Weeks before symptom** | **Aphid transmissibility (No. plants infected /No. plants inoculated)** |
| --- | --- | --- | --- | --- |
| WT | 19/20 | 19 | 3.3±0.5 | 20/20 |
| Mut-∆5670 | 19/20 | 0 | N* | 1/20 |
| Rev-85nt | 20/20 | 20 | 3.8±0.5 | 20/20 |
| Rev-8AA | 19/20 | 19 | 3.6±0.3 | 20/20 |

* (N: no symptom)
