# Supplementary material for: An aromatic amino acid and associated helix in the C-terminus of the potato leafroll virus minor capsid protein regulate systemic infection and symptom expression
Source: PLoS Pathog. 2018 Nov 15;14(11):e1007451. doi: 10.1371/journal.ppat.1007451 (PMC6264904; doi:10.1371/journal.ppat.1007451)
Supplement: S3 Table — (DOCX) [file ppat.1007451.s011.docx]

**S3 Table. Pathogenicity of PLRV RTD N-terminal deletion mutants infecting hairy nightshade plants.** Our recently published paper in Journal of Virology (e01544-17. doi: 10.1128/JVI.01544-17) constructed two mutants (Mut12 and Mut13) that a large deletion in the N-terminus of the RTD but the regions required for RTP translation were reserved. Then we infiltrated these two mutants into hairy nightshade in this research, the interveinal symptom was observed and as shown in the table, indicating the N-terminus of the PLRV RTD that do not interfere with RTP translation has no effect on symptom expression. The details of Mut12 and Mut13 can be found in Journal of Virology (e01544-17. doi: 10.1128/JVI.01544-17).

| **PLRV/mutants** | **No. plants infected/No. plants agroinfiltrated** | **No. plants with symptom** | **Weeks before symptom** |
| --- | --- | --- | --- |
| WT | 15/15 | 15 | 3.3±0.5 |
| Mut12 | 15/15 | 15 | 5±0.5 |
| Mut13 | 15/15 | 15 | 5.8±0.5 |
